# Inter-Specific Genetic Exchange Despite Strong Divergence in Deep-Sea Hydrothermal Vent Gastropods of the Genus *Alviniconcha*

**DOI:** 10.3390/genes13060985

**Published:** 2022-05-31

**Authors:** Jade Castel, Stéphane Hourdez, Florence Pradillon, Claire Daguin-Thiébaut, Marion Ballenghien, Stéphanie Ruault, Erwan Corre, Adrien Tran Lu Y, Jean Mary, Pierre-Alexandre Gagnaire, François Bonhomme, Corinna Breusing, Thomas Broquet, Didier Jollivet

**Affiliations:** 1 ‘Dynamique de la Diversité Marine’ (DyDiv) Lab, Station Biologique de Roscoff, Sorbonne Université, CNRS, UMR 7144, Place G. Teissier, 29680 Roscoff, France; daguin@sb-roscoff.fr (C.D.-T.); marion.ballenghien@sb-roscoff.fr (M.B.); stephanie.ruault@sb-roscoff.fr (S.R.); jmary@sb-roscoff.fr (J.M.); thomas.broquet@sb-roscoff.fr (T.B.); jollivet@sb-roscoff.fr (D.J.); 2 Laboratoire d’Ecogéochimie des Environnements Benthiques, Observatoire Océanologique de Banyuls, Sorbonne Université, CNRS, UMR 8222, Avenue Pierre Fabre, 66650 Banyuls-sur-Mer, France; stephane.hourdez@obs-banyuls.fr; 3 Unité Biologie et Ecologie des Ecosystèmes Marins Profonds, Université de Brest, Ifremer, CNRS, 29280 Plouzané, France; florence.pradillon@ifremer.fr; 4 ABiMS Bioinformatics Facility, Station biologique de Roscoff, Sorbonne Université, CNRS, FR2424, Place G. Teissier, 29680 Roscoff, France; corre@sb-roscoff.fr; 5 Team MBE, Department Genφ, ISEM, Université de Montpellier, CNRS, EPHE, IRD, 34110 Montpellier, France; adrien.tran-lu-y@etu.umontpellier.fr (A.T.L.Y.); pierre-alexandre.gagnaire@umontpellier.fr (P.-A.G.); francois.bonhomme@umontpellier.fr (F.B.); 6 Graduate School of Oceanography, University of Rhode Island, 215 South Ferry Rd, Narragansett, RI 02882, USA; cbreusing@uri.edu

**Keywords:** speciation, secondary contact, nuclear and mitochondrial genome, transcriptome, DILS

## Abstract

Deep hydrothermal vents are highly fragmented and unstable habitats at all temporal and spatial scales. Such environmental dynamics likely play a non-negligible role in speciation. Little is, however, known about the evolutionary processes that drive population-level differentiation and vent species isolation and, more specifically, how geography and habitat specialisation interplay in the species history of divergence. In this study, the species range and divergence of *Alviniconcha* snails that occupy active Western Pacific vent fields was assessed by using sequence variation data of the mitochondrial *Cox1* gene, RNAseq, and ddRAD-seq. Combining morphological description and sequence datasets of the three species across five basins, we confirmed that *A. kojimai*, *A. boucheti*, and *A. strummeri*, while partially overlapping over their range, display high levels of divergence in the three genomic compartments analysed that usually encompass values retrieved for reproductively isolated species with divergences rang from 9% to 12.5% (mtDNA) and from 2% to 3.1% (nuDNA). Moreover, the three species can be distinguished on the basis of their external morphology by observing the distribution of bristles and the shape of the columella. According to this sampling, *A. boucheti* and *A. kojimai* form an east-to-west species abundance gradient, whereas *A. strummeri* is restricted to the Futuna Arc/Lau and North Fiji Basins. Surprisingly, population models with both gene flow and population size heterogeneities among genomes indicated that these three species are still able to exchange genes due to secondary contacts at some localities after a long period of isolation.

## 1. Introduction

The tectonic history and fragmentation along oceanic ridges of deep-sea hydrothermal ecosystems provide an interesting opportunity to study the evolution of divergence and speciation. Since the discovery of the first faunal communities associated with hydrothermal vents on the Galapagos rift in the late 1970s [1], many endemic species have been described. However, because sampling in the deep sea is difficult, our understanding of the evolutionary and ecological mechanisms that have led to the present-day distribution of vent-associated fauna is still relatively limited. The continuous movement of the Earth’s tectonic plates since the formation of the oceans has acted as an evolutionary force to partition the vent fauna over geological time scales [2,3,4]. Tectonically driven allopatric speciation, thus, appears to be common in hydrothermal systems [5]. Hydrothermal vents are fragmented not only in space but also in time, as tectonic movements and volcanism lead to the recurrent birth and extinction of vent sites. These dynamics are likely to lead to extinction/recolonisation events in the vent populations, which in turn promotes either population isolation or secondary contact [6]. In addition, thermal and chemical conditions (high temperature, low oxygen, high concentration of CO_2_, H_2_S, H_2_, and CH_4_ [7]) may induce strong purifying selection on the molecular arsenal to adapt to this hypoxic and highly toxic environment [8] and could, thus, favour morphological and functional stasis. Despite the homogenising and convergent effect of living under sulfidic conditions, mineral effluents could vary locally depending on the nature of the oceanic crust and associated fluid chemistry [9,10] and should affect the distribution, interactions, and speciation mechanisms of the species present. A large number of hydrothermal species develop symbiosis with microorganisms. In this case, environmental factors may also act on microbiomes, where interactions between host and microbiota can be affected and participate in local adaptation.

As part of deep-sea hydrothermal vent communities, large symbiotic gastropods of the genus *Alviniconcha* (Gastropoda: Abyssochrysoidea) inhabit warm (7–42 °C), sulphur-rich (250 µM), and poorly oxygenated (<50 µM) diffuse venting environments where they represent a group of engineer species [11,12]. To date, six species of *Alviniconcha* have been identified on the basis of their high level of genetic divergence of the mitochondrial gene *Cox1* [13]. Assuming that the evolutionary rate of the *Cox1* gene has been constantly low (around 0.0015 substitutions per site and million years), the speciation events that led to the current species are thought to have occurred between 38 and 10 million years ago [14]. This suggests a long history of allopatric speciation that may be linked to the plate history of the region and variations of environmental conditions that could have modified the nature of symbiotic interactions.

Isolating mechanisms that limit the exchange of genetic material between populations become stronger as lineages diverge [15,16]. Moreover, the mitochondrial genome evolves generally faster than the nuclear genome due to differences in selective pressures (e.g., purifying selection and selective sweeps), nuclear compensation [17], and the speed at which haplotypes are sorted during the initial part of the isolation process with a fourfold smaller effective population size when sex ratio is unbiased. This difference promotes mito-nuclear incompatibilities [18,19], which often create post-zygotic barriers during the speciation process [20,21]. As a consequence, most species in the speciation phase that are still capable of hybridisation display relatively low divergence, typically no more than a few percent in the mitochondrial genome [22,23] and even less in the nuclear genome [24]. One can, therefore, expect the six *Alviniconcha* species to be strongly isolated. However, the species of *Alviniconcha* have so far been distinguished solely on the basis of two mitochondrial markers (*Cox1* and 16S) and a few slowly-evolving nuDNA genes (18S, 28S). We lack the genome-wide estimates of diversity and divergence that are necessary to confront nuclear and mitochondrial genetic patterns and infer the evolutionary history of these species. This is particularly important for marine invertebrates considering that they often form complexes of cryptic but highly divergent species (*Cox1* divergence > 15%) that are nevertheless still capable of hybridising locally with porous nuclear genomes [25,26,27].

Here, we focus on the species *A. kojimai*, *A. boucheti*, and *A. strummeri*, which are found in back-arc basins from the Western Pacific Ocean [13]. *A. kojimai* and *A. boucheti* partially overlap in the Manus, North Fiji, Futuna, and Lau back-arc basins, and *A. strummeri* [13] is currently restricted to the most southern part of the Lau Basin and the Futuna volcanic Arc [13,14,28]. Like many hydrothermal species, *Alviniconcha* snails have long been considered cryptic (i.e., species that cannot be separated on the basis of their morphology but that are genetically distinct) [13,29,30]. However, the recent study of Laming et al. [28] described morphological and anatomical differences between the three above species in the active hydrothermal zone near to the volcanic arc of Wallis and Futuna.

Our study based on three species of *Alviniconcha* aims to (1) describe the current distribution of *A. kojimai*, *A. boucheti*, and *A. strummeri* in the western Pacific back-arc basins, (2) estimate their genetic divergence on different genomic datasets, mtDNA, nuDNA, and transcribed sequences (i.e., transcriptomes) to test whether these divergences are congruent and proportional to the time elapsed since estimated species separation, (3) understand the demographic history of these species and whether they have diverged in allopatry, and (4) investigate whether these species display fixed morphological differences over their overall species range (i.e., five western Pacific back-arc basins) using morphological traits previously highlighted as diagnostic in the volcanic arc of Wallis and Futuna [28].

## 2. Materials and Methods

### 2.1. Sampling

**Figure 1:**
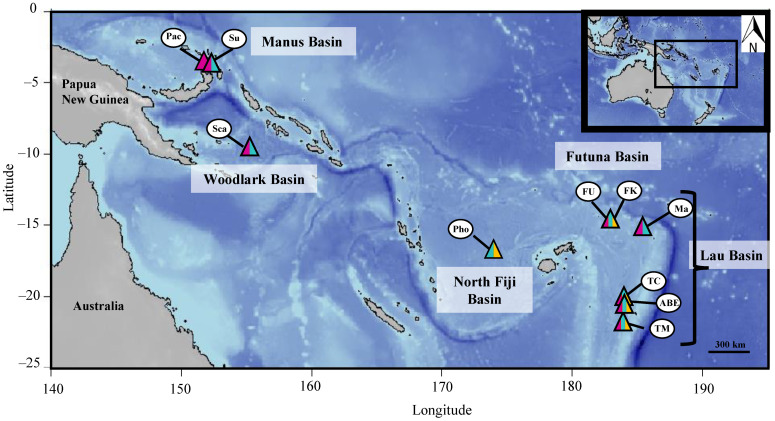
Sampled localities of *Alviniconcha* species during the Chubacarc expedition. Manus Basin: Susu (Su); Pacmanus (Pac). Woodlark Basin: Scala (Sca). North Fiji Basin: Phoenix (Pho). Futuna Arc: Fatu-Kapa (FK); Fati-Ufu (FU). Lau Basin: Mangatolo (Ma); Tow Cam (TC); ABE (ABE); Tu’i Malila (TM). Colours indicate species occurrence: purple, *A. boucheti*; turquoise, *A. kojimai*; yellow, *A. strummeri*. The map background was obtained using the R package marmap [31].

A total of 816 individuals of *Alviniconcha* spp. were sampled during the Chubacarc expedition (https://campagnes.flotteoceanographique.fr/campagnes/18001111/ accessed on 10 February 2022) conducted in May–June 2019 at 18 different vent fields from five back-arc basins of the Western Pacific Ocean on board the N/O L’Atalante (chief scientists: S. Hourdez and D. Jollivet). Species were identified a posteriori following a barcoding approach using the mitochondrial *Cox1* gene (see results, Table S1 and Figure 1) and the diagnostic reference sequences of Johnson et al. [13]. Sampling was conducted with the tele-manipulated arm of the remotely operated vehicle (ROV) Victor6000. The snails were scooped either on diffuse venting sites or on the wall of active chimneys and transferred into biological boxes (“bioboxes”). Upon recovery on board, samples were temporarily stored in tanks containing refrigerated sea water (4 °C) before being examined and dissected. Several soft tissues (gill, foot, mantle) were preserved in both 80% ethanol and RNALater, and fresh foot tissue was also used for immediate DNA extraction on board (see below). Geographic information about the gastropod collection used during this study is summarised in Table S1.

Ten frozen (−80 °C) individuals from the TN 235 Lau expedition (2009) on board the R/V Thomas G. Thompson (chief scientist: C.R. Fisher) were used for total RNA extraction in order to produce RNAseq datasets and subsequent transcriptome assemblies.

### 2.2. Morphological Analysis

Prior to the dissection of the animals, the shell was photographed, and a series of six distinct shell traits (length, width, spires, and aperture lengths, as detailed in Chiu et al. [32] and in Figure S1) were measured with a calliper. Because specimens within and between collections had different sizes, we used the total shell length to standardise the value of the remaining five traits. After checking there was no growth allometry for these five traits, they were standardised so that they all had a mean of 0 and a standard deviation of 1. These five transformed variables were then analysed in a principal component analysis (PCA) and a linear discriminant analysis (LDA). All analyses were performed in R using functions from the packages ade4 [33] and MASS [34].

**Figure 2:**
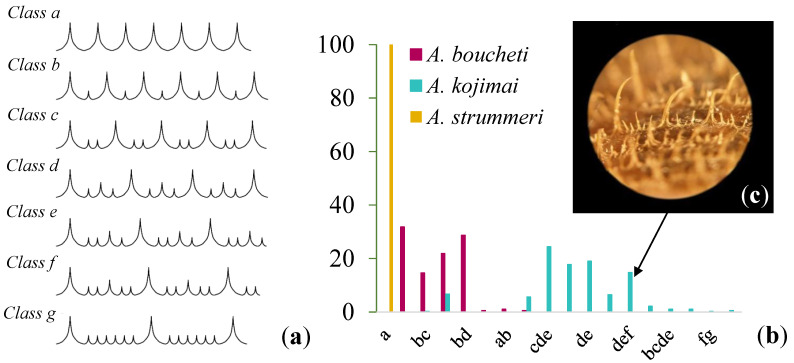
(**a**) Arrangement of small, medium, and large bristles on *Alviniconcha* shells; (**b**) percentage of bristles arrangement categories observed in each *Alviniconcha* species. Different categories of bristle arrangements may be encountered on the shell of a given specimen depending on the row considered, which are noted ab, bcd, etc. to account for such heterogeneity; (**c**) example of bristle arrangement for an individual of *Alviniconcha kojimai*.

A shell fragment (approximately 4 × 3 cm) was cut off near the shell aperture in a subset of 732 individuals to evaluate the ‘hairy’ periostracal ornamentation. It consists of bristles arranged in rows. Bristles were classified into three different types: small, medium and large, which have been used to discriminate species [28]. These three types of bristles were arranged in seven different ways (noted a–g) for a given row (Figure 2a). Because each individual may bear several arrangements depending on the examined rows, individuals were characterised according to the different bristle arrangements they had (e.g., ab, abc, etc.). The presence of either a double- or single-twisted columella was also observed and recorded for each individual [28].

### 2.3. Mitochondrial Cox1 Sequence Analysis

DNA was extracted on board from a small piece of fresh foot tissue of the 816 individuals using either the NucleoSpin^®^ Tissue kit (Macherey-Nagel, Karlsruhe, Germany) or a modified CTAB protocol [35,36] for the amplification of the mitochondrial *Cox1* gene and the preparation of ddRAD libraries. Genomic DNA quality was checked following a 1% agarose gel electrophoresis, and DNA extracts were quantified with a fluorometric method using the Quantifluor Promega kit in a Spark plate reader (Tecan). A 709 bp fragment of the mitochondrial cytochrome *c* oxidase subunit I (*Cox1*) gene was amplified from diluted DNA extracts with the LCO1490 (5′–GGTCAACAAATCATAAAGATATTGG–3′) and HCO2198 (5′–TAAACTTCAGGGTGACCAAAAAATCA–3′) primer pair [37]. PCR amplifications were conducted in a final volume of 25 µL using 2.5 µL of diluted template DNA, 0.1 µM of each primer, 50 µM of each dNTP, 2 mM MgCl_2_, 0.5 U of Flexi GoTaq^®^ polymerase (Promega), and 0.1 mg/mL bovine serum albumin in 1× Green GoTaq^®^ Reaction Buffer (Promega). The amplification protocol was as follows: 94 °C for 3 min, 35 cycles with 94 °C for 30 s, 52 °C for 45 s, 72 °C for 1 min, and a final elongation step at 72 °C for 5 min, in a T100 thermocycler (Biorad). Because of the co-amplification of an additional nonspecific small fragment for some specimens with the forward primer, PCR products were sequenced only from the HCO2198 primer, using the Sanger method (Eurofins Genomics, Ebersberg, Germany). The *Cox1* gene sequences were manually checked for polymorphic sites using CodonCode aligner (v. 5.1.5, Codon Code Corporation), edited in Bioedit [38], and aligned by ClustalX [39]. One reference sequence representing each of the described *Alviniconcha* species from Johnson et al. [13] and Suzuki et al. [40] (Genbank accession numbers: AB235216; KF467675; KF467921; KF467741; KF467873; KF467896) was added to the dataset. Sequences of five individuals of the species *Ifremeria nautilei* also collected during the Chubacarc cruise were used as an outgroup. Haplotype and nucleotide diversities (*Hd* and *π,* respectively), the substitution rates at non-synonymous (*d*_N_) and synonymous sites (*d*_S_), and the absolute population divergence (*d*_XY_) were estimated with DnaSP 6.0 [41,42] between the three pairs of *Alviniconcha* species. McDonald–Kreitman tests were used to evaluate the degree of adaptive evolution of the *Cox1* gene within and between species. Inter-specific pairwise *F*_ST_ was calculated from haplotype frequencies with ARLEQUIN V.3.5 [43]. A haplotype network was created with the median-joining method [44] using the software PopArt [45] to depict phylogenetic relationships on the basis of the mitochondrial sequences. 

### 2.4. Nuclear Genome Analysis

#### 2.4.1. ddRAD Library Preparation

Foot tissue fragments from 570 individuals were used for the production of individual double digest restriction-associated DNA (ddRAD) libraries [46,47] following the protocol fully detailed in Daguin-Thiébaut et al. [48]. Briefly, genomic DNA of each individual (~60 ng) was digested with the restriction enzymes *Pst*I and *Mse*I, ligated to Illumina Truseq adapters containing a 6 bp barcode, and purified with AMPure XP beads (Beckman Coulter) prior to PCR amplification with Illumina indexed primers. Individual PCR products were checked on agarose gels and then pooled in three distinct groups of multiplexed individuals (24 barcodes and eight Illumina indices) before performing a final fragment size selection (300–800 bp) using a Pippin Prep system (Sage Science). Distributions of DNA fragment sizes were checked in a high-sensitivity dsDNA chip using a BioAnalyzer 2100^TM^ instrument (Agilent). The three pools were sent to Novogene Europe (Cambridge, UK) for 150 bp paired-end sequencing on an Illumina Novaseq6000 sequencer.

#### 2.4.2. Bioinformatic Filtering of Illumina Reads

Raw reads were first demultiplexed using the process_radtags module of the Stacks software version 2.52 [49,50] that also removed adapters and low-quality reads. Average sequence quality per read and GC content were checked using multiQC version 1.7 [51]. Paired-end 144 base reads were assembled using the de novo pipeline in Stacks v2.52 [49,50]. Assembly parameters (*m*: 6; *M*: 14; *n*: 14) were chosen after empirical testing over a range of values (*m*: 2–6; *M*: 2–12; *n*: 2–18) on a subset of 23 individuals that included the three *Alviniconcha* species. Among these 23 individuals, 13 were triplicated for a total of 49 samples, which made it possible to evaluate the genotyping error rate according to the parameters tested. The applied parameter settings resulted in the greatest number of loci retained while maintaining a minimum genotyping error rate (see details in Supplementary Materials Figure S2). Trimmed reads were aligned in unique stacks (RAD-seq equivalent of alleles) if six or more identical reads were found within an individual (*m* = 6). Alleles were then compiled within each individual into unique loci if they differed by less than 14 nucleotides (~10% divergence; *M* = 14) to consider the divergence of putative introgressed alleles between species. To assess the inter-specific divergences and shared polymorphisms, loci were then assembled across all individuals into homologous loci if they differed by less than the same number of nucleotide changes (~10%, *n* = 14). A high value of *n* was chosen, despite the risk of assembling paralogues, because previous studies showed a strong divergence between these species [13,14]. To identify single-nucleotide polymorphism sites (SNP), clustering was performed with the denovo_map pipeline of Stacks-2.52, using a popmap of 570 individuals and the parameters defined previously [49,50]. First, a sub-popmap consisting of 150 individuals distributed among the three species (one species constituting one population) and the different basins sampled was used for the construction of the inter-individual catalogue (ctacks). Second, all individuals were mapped to this catalogue (sstacks). The raw SNP data were filtered against the following thresholds: minimum individuals sharing a locus in a population *r* ≥ 0.8 and minor allele count MAC ≥ 4. Using this first dataset (hereafter referred to as the “raw” dataset), we examined the number of RAD loci and SNPs that were genotyped within each species or were shared between species, to get a picture of the impact of divergence between species in the RAD-seq dataset.

The SNPs identified by Stacks were then further filtered for missing data using R scripts to only keep the SNPs genotyped in 90% of the individuals and to keep only the individuals that were genotyped at more than 85% of the SNPs. In the end, a high-quality dataset of 60,084 SNPs for 498 individuals (250 *A. kojimai*, 212 *A. boucheti* and 36 *A. strummeri*) was obtained.

#### 2.4.3. Population Structure, Divergence, and Admixture

The population structure associated with the SNPs shared between the three *Alviniconcha* species was visualised in R using a principal component analysis (PCA; adegenet package [52]). The population divergence (*d*_XY_) between species, the nucleotide diversity (*π*), and the net divergence (*d*_A_ = *d*_XY_ − (*π*_X_ + *π*_Y_)/2) were estimated following Nei and Li [42] using the fstat option in Stacks-2.52. *F*_ST_ between species and between basins within species was calculated with the R package Hierfstat [53]. Individual admixture coefficients were estimated using the function snmf from the R package LEA [54]. Assuming *K* ancestral populations, the R function snmf provides least-square estimates of ancestry proportions for all analysed individuals [55]. This analysis was carried out in 20 runs with the 498 individuals obtained previously during the ddRAD-seq analysis and *K* = 3 (derived from PCA analyses and the best entropy value, see results and Figure S10). 

#### 2.4.4. Demographic Inference

The ABC software DILS (Demographic Inferences with Linked Selection by using ABC [56]) was used to determine which historical scenario of isolation versus gene flow might best explain the genetic structure observed today between the three *Alviniconcha* species in the Western Pacific ocean. In an approximate Bayesian computational framework, DILS compares summary statistics from simulated and observed datasets to identify plausible demographic scenarios and to jointly estimate population parameters such as effective population sizes, time of isolation, and past and contemporary gene flow between populations. It also takes into consideration changes in population size, local selective effects along the genome (variation in the effective size *N*_e_ among loci), and semipermeable barriers to gene flow (variation in the effective migration rate *me* among loci). Because DILS is designed to simulate divergence scenarios for a pair of populations, we reanalysed our RAD-seq dataset for the three pairs of species by running the Stacks *population* function using the common catalogue of loci described above, with *r* = 0.8 and MAC = 4. Then, we only kept SNPs genotyped in at least 90% of the individuals. For the demographic inference, 5655 RAD loci and 265 individuals were used for the *A. boucheti*/*A. strummeri* pair, 8808 RAD loci and 495 individuals were used for the *A. kojimai*/*A. boucheti* pair, and 5775 RAD loci and 304 individuals were used for the *A. kojimai*/*A. strummeri* pair. Because DILS uses a random subsample of 1000 loci to estimate observed summary statistics, we ran 10 independent analyses for each pair of species, under the following main models: strict isolation (SI), ancient migration (AM), isolation with migration (IM), and secondary contact (SC). During these runs, the method accounted for two events of instantaneous population size change (one when the ancestral population splits into two daughter populations and one at any later time). Finally, for each run, the program also tested whether *N*_e_ and *me* are distributed heterogeneously along the genome (therefore accounting for the effect of linked selection and local barrier effects on gene flow along the genome). The goodness of fit of each run was estimated using the Euclidean distance between the observed and simulated summary statistics calculated with an accompanying Python script. Detailed parameter settings are provided in the Supplementary Materials (see example of detailed parameter settings section). 

### 2.5. Transcriptome Analysis

Total RNA was extracted using the NucleoSpin^®^ RNA Plus kit (Macherey-Nagel) according to the manufacturer’s protocol from the frozen gill tissue of two *A. kojimai*, two *A. strummeri*, and three *A. boucheti* individuals. RNA integrity was confirmed using a 2100 Bioanalyzer (Agilent Technologies, Palo Alto, CA, USA). RNA extracts were sent to Genome Québec (Montréal, QC, Canada) for RNAseq library construction and sequencing on half a lane of Illumina Novaseq6000 in PE150. The raw reads were cleaned by removing adaptor sequences, empty reads, and low-quality sequences (including reads with unknown sequences ‘N’) with Trimmomatic [57]. A taxonomic assignment was made with Kraken v.2 [58] from the reads to only keep the eukaryotic reads for the assembly. This taxonomic-based clean-up was conducted because gills of our target species contained large amounts of endosymbiotic bacteria whose RNA was not totally discarded from the polyA hybridisation technique associated with the library preparation. Following the Kraken filtering, around 5–10% of the initial reads of each individual were assigned to prokaryotes. The 90–95% remaining reads were then assembled in rnaSPAdes 3.13.1 using default parameters [59]. The quality of the assembly was checked by looking at the remapping rate and by performing a Busco analysis. After sequence assembly, the resulting contigs were used in the Galaxy pipeline AdaptSearch (repository https://github.com/abims-sbr/adaptsearch accessed on 10 February 2022 ) to find orthologous sequences between species, estimate species divergence from the coding sequences, and find genes under positive selection. The first step of this pipeline is to filter transcripts to only keep one sequence per gene using both the length and the quality score of the transcripts and to perform an additional CAP3 assembly [60]. Transcripts of the different species were then put together in orthogroups with Orthofinder [61]. The sequences of orthogroups were aligned using BlastAlign [62], and the reading frame was identified from the species alignment with the in-house Python script CDSsearch (in the AdaptSearch suite) using Blastx against NCBI libraries. Finally, gene alignments were concatenated over the whole shared transcriptome and the alignment was used to reconstruct a phylogenetic tree with RAxML [63]. This alignment of coding sequences was then used to estimate the standard diversity index (*π*) within species and the absolute divergence (*d*_XY_) between species using DnaSP 6.0 [41,42]. The divergence accumulated at non-synonymous (*d*_N_) and synonymous sites (*d*_S_) along terminal branches, and their ratio was calculated with CodeML [64]. The net number of nucleotide substitutions per site between populations (*d*_A_) was estimated from *d*_XY_ and the values of the nucleotide diversity (*π*) found within each species from 2–3 individuals.

## 3. Results

### 3.1. Species Distribution

The *Cox1* barcode assignment allowed us to get a better understanding of the distribution range for each species (Figure 1). *Alviniconcha kojimai* was the most abundant species sampled during the 2019 Chubacarc expedition. It was present throughout the study area (with the exception of the Pacmanus field (Manus)) and dominated over the two other *Alviniconcha* species in the Futuna Arc, and the Lau, and North Fiji Basins where it was present in 15 out of 18 population samples (Figure 1, Table S1). *A. strummeri*, found in only seven population samples from the Futuna Arc and the Lau and North Fiji Basins, was the most geographically restricted species. It was always found with *A. kojimai* and, on the basis of visual clues, was typically found at the periphery of snail patches. *A. boucheti* was sampled at all locations except the North Fiji Basin (Figure 1; Table S1). The species dominated *Alviniconcha* assemblages in the Manus and Woodlark Basins where it represented 71% of the samples (present in eight population samples) and was also the only species found in the PacManus field. Although mixed with *A. kojimai* at some locations, *A. boucheti* was found preferentially (in 60% of the samples; Table S1) on the wall of active hydrothermal chimneys, as previously suggested in Beinart et al. [65]. 

### 3.2. Morphology

We measured six shell traits from 700 *Alviniconcha* individuals. The distribution of the values of shell traits is shown in Supplementary Figure S3. All measurements were significantly different between species (ANOVA, *p* < 0.001), with *A. boucheti* having on average a larger shell (at all measured traits) than *A. kojimai*, and *A. strummeri* being the smallest species. Only a small fraction of the variance in shell traits seemed to be related to species identity (see PCA axis 2 in Figure S5). This was due to the low power in discriminating species using solely shell dimensions measured (see LDA in Figure S7). Since the reclassification of species using the whole dataset was correct for only 75% of the individuals (and below 2% for *A. strummeri* in particular), we did not further evaluate the capacity of the LDA using training and test subsets of the data. There could be some differences in shell morphology associated with geography (as suggested by shell size distributions, Figure S4, and PCA axis 2 in Figure S6) but further analyses will be required to refine these results and investigate potential effects of the environment (e.g., position of snails in diffusion zones or chimneys).

By contrast, the shell ornamentation of bristles was clearly distinct between species except for a slight overlap between *A. kojimai* and *A. boucheti* (Figure 2). *A. strummeri* was characterised by only one category of bristles (type a) corresponding to only long and uniformly sized spikes. *A. boucheti* was more polymorphic with several categories of rows (ab, b, bc, bcd, bd, and abc) corresponding to rows showing repetitions of 0–3 smaller bristles between two long ones. Lastly, *A. kojimai* was the most polymorphic species in terms of row categories but with a shifted number of smaller bristles ranging from two to six between two subsequent long ones. Despite this heterogeneous arrangement of bristles in *A. boucheti* and *A. kojimai*, periostracal ornamentation only overlapped at the bristle arrangements bcd and d between these two species (Figure 2b), which characterised 30 *A. kojimai* and 43 *A. boucheti* individuals over their whole geographic range.

In addition, our morphological observation of the three species also indicated that *A. boucheti* was the only species displaying a single twisted columella as opposed to *A. kojimai* and *A. strummeri* which exhibited a double twisted columella, as previously reported by Laming et al. [28] for individuals from the volcanic arc of Wallis and Futuna.

### 3.3. Mitochondrial Cox1 Gene Analysis

**Figure 3:**
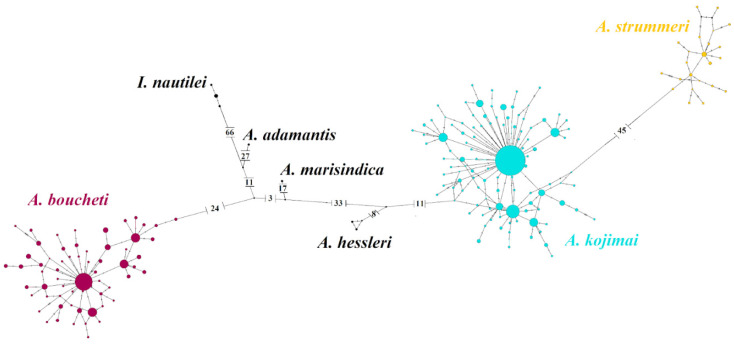
Haplotype network of 722 individuals of *Alviniconcha* spp for *Cox1* partial mitochondrial sequence rooted with *Ifremeria nautilei* (*n* = 4). Circles represent individual haplotypes, while circle size is proportional to haplotype frequency. The numbers on the branches indicate the number of mutations between haplotypes. Purple: *A. boucheti*; turquoise: *A. kojimai*, and yellow: *A. strummeri*.

An alignment of 599 bp was obtained for 722 individuals of *Alviniconcha* spp. for the mitochondrial *Cox1* gene. Haplotype diversity (*Hd*) ranged from 0.8 to 0.96 across species while nucleotide diversities (*π*) ranged from 0.003 to 0.008 (Table 1). The mitochondrial *Cox1* haplotype network (Figure 3 and Figure S8) indicated that the three studied *Alviniconcha* species display complete mitochondrial lineage sorting over their whole geographic range, as previously reported [13,14]. The net divergence *d*_A_ was smallest between *A. kojimai* and *A. strummeri* (*d*_A_ = 8.6%), while the two other species pairs displayed *d*_A_ values 1.4 times greater (*d*_A_ = 11.8% and 12.3%; Table 2). Genetic differentiation as measured by *F*_ST_ between species pairs was very strong (between 0.84 and 0.92, Table 2). The *d*_N_/*d*_S_ ratios estimated between pairs of species were low, with values ranging between 0.003 and 0.014. The McDonald–Kreitman test performed between the three pairs of species was not significant, suggesting that this gene evolved neutrally during the separate evolution of the three species. 

### 3.4. ddRAD-seq Analysis along the Nuclear Genome

**Figure 4:**
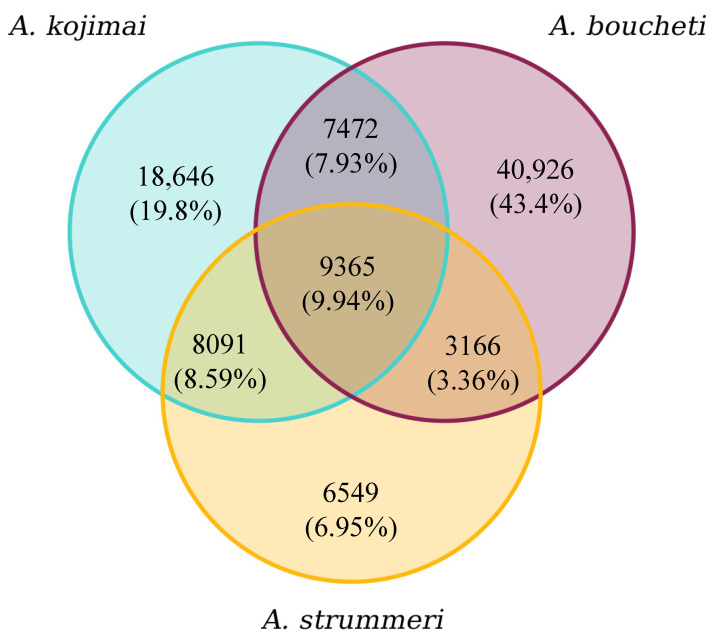
Number of RAD loci sequenced in each species (under the condition that a locus is considered in any species if it is sequenced in at least 80% of the individuals of that species). There were 94,215 RAD loci overall (640,002 SNPs). The 9365 loci shared among all species were then further filtered to produce the final dataset used in downstream analyses.

The “raw” dataset (where an SNP is retained if genotyped in at least 80% of individuals within any species) contained 640,002 SNPs from 94,215 RAD loci. The distribution of these loci among species is shown in Figure 4. Almost 76% of the RAD loci sequenced in *A. strummeri* were also sequenced in at least one of the two other species, while this fraction was 57% in *A. kojimai* and 33% in *A. boucheti.*

**Figure 5:**
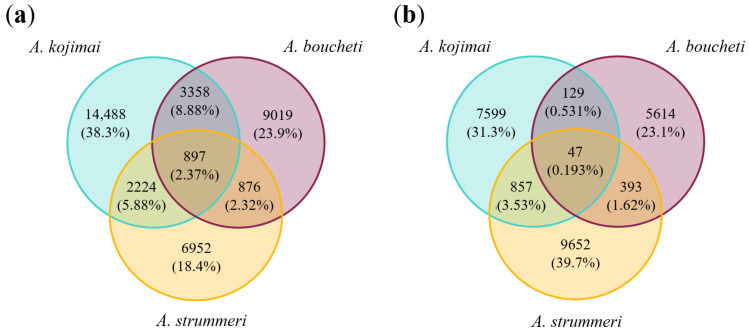
Distribution of polymorphism in the 60,084 SNPs shared by *A. kojimai* (*n* = 250), *A. boucheti* (*n* = 212), and *A. strummeri* (*n* = 36). In panel (**a**), the numbers exclusive to each circle show the number of SNPs that are polymorphic within one species and fixed in the two others (for instance, there are 9019 SNPs that are polymorphic within *A. boucheti* only). Panel (**b**) shows the same data but considering a SNP as polymorphic only if the allelic frequency of either allele was above 0.0139 (minimum allele frequency observable in *A. strummeri*, the species with the smallest sample size).

Further filtering on missing data of the 9365 loci in common across the three species led to a final dataset containing 60,084 biallelic SNPs on 4380 RAD loci genotyped in 498 individuals (triplicated removed). Among these SNPs, 80% were polymorphic within a single species while being fixed (differentially or not) in the two others (Figure 5a). Only 897 SNPs (2.4%) were polymorphic in all three species. However, these numbers strongly depend on sample size. With only 36 *A. strummeri* individuals genotyped, the smallest measurable allelic frequency at any SNP in this species was 0.0139, while it was much smaller for the two other species, which had larger sample sizes. Looking at the distribution of polymorphisms after reclassifying the SNPs as polymorphic only if they had a minimum allelic frequency of at least 0.0139, we can confirm that there is more polymorphism shared between *A. strummeri* and *A. kojimai* than between *A. boucheti* and either species (Figure 5b).

**Figure 6:**
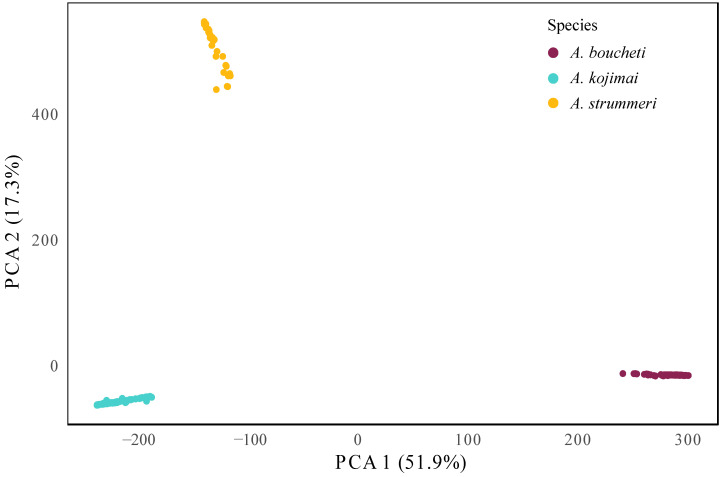
Principal component analysis of multilocus genotypes using 60,084 SNPs across 498 individuals of *Alviniconcha* spp. Purple: *A. boucheti*, turquoise: *A. kojimai*, and yellow: *A. strummeri*.

**Figure 7:**
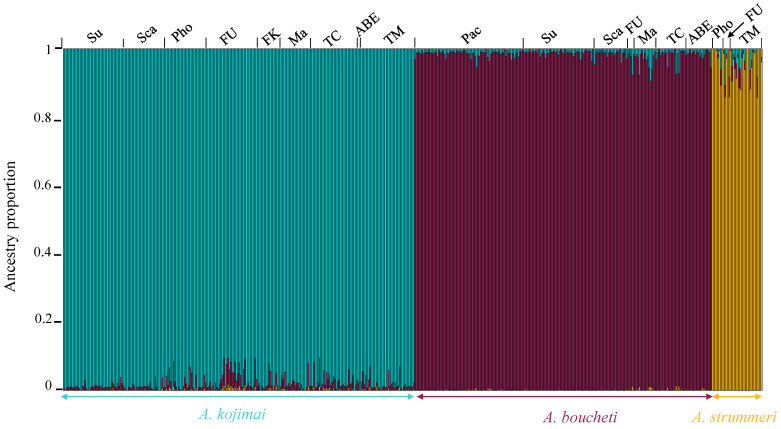
Ancestry coefficients bar plots representing *K* = 3 for the three species of *Alviniconcha* sampled during the Chubacarc expedition. This graphic was obtained using the snmf function in the package LEA (R software [55]) on 60,084 SNPs for 498 individuals with 20 runs. Each vertical bar corresponds to one individual. The colours (purple: *A. boucheti*, turquoise: *A. kojimai*, and yellow: *A. strummeri*) were assigned according to the ancestry inferred for each cluster. Manus Basin: Susu (Su); Pacmanus (Pac). Woodlark Basin: Scala (Sca). North Fiji Basin: Phoenix (Pho). Futuna volcanic Arc: Fatu-Kapa (FK); Fati-Ufu (FU). Lau Basin: Mangatolo (Ma); Tow Cam (TC); ABE (ABE); Tui Malila (TM).

The nucleotide diversity (*π*) was the same between species even though the geographic distribution of *A. strummeri* was more restricted (0.0013 in *A. kojimai* and 0.0014 in *A. strummeri* and *A. boucheti*) (Table 1). The PCA analysis using all 60,084 SNPs clearly separated the three species of *Alviniconcha* along the first two principal components (explaining 51.9% and 17.3% of the total variance, respectively) (Figure 6). The net divergence (*d*_A_) was 1.8% between *A. kojimai* and *A. strummeri* and about 1.6 times greater (3.1%) between the two other pairs of species. The total genetic differentiation (as estimated by *F*_ST_) between populations of *A. kojimai* and *A. strummeri* was high (0.84), but lower than the nearly fixed *F*_ST_ values obtained for the two other species pairs (Table 2). These values were close to one as expected for species having nearly completed their allelic lineage sorting (Table 2; Figure S9), in sharp contrast to the global level of differentiation between basins within each species (*F*_ST_ = 0.003 in *A. boucheti*, 0.01 in *A. kojimai*, and 0.019 in *A. strummeri*; see also Table S2 for pairwise values between populations of the five back-arc basins). The admixture analysis clearly separated the three *Alviniconcha* species, in agreement with the PCA analysis and results from the mitochondrial *Cox1* gene (Figure 3 and Figure 6). The admixture bar plot (Figure 7), however, still exhibited traces of shared polymorphism or introgressed alleles between the three species. Indeed, some individuals displayed up to 15% of hetero-specific ancestry (especially in *A. strummeri*). In *A. strummeri*, individuals with the highest hetero-specific ancestry were collected at both Futuna and Lau but not at the Phoenix site on the North Fiji triple junction. In *A. kojimai* and *A. boucheti*, individuals with the highest hetero-specific ancestry were also retrieved along the Futuna volcanic Arc and the Lau Basin, where the two species are found in syntopy, with *A. kojimai* from Futuna being 2.3 times more hetero-specific than the others. However, this pattern was not observed on the Pual ridge of the Manus Basin (Pacmanus, Fenway, Big Papi) where only *A. boucheti* is found. The difference in the proportion of hetero-specific ancestries between basins was, however, not sufficient to affect pairwise *F*_ST_ values between basins for each of the three species (see Table S2). 

**Figure 8:**
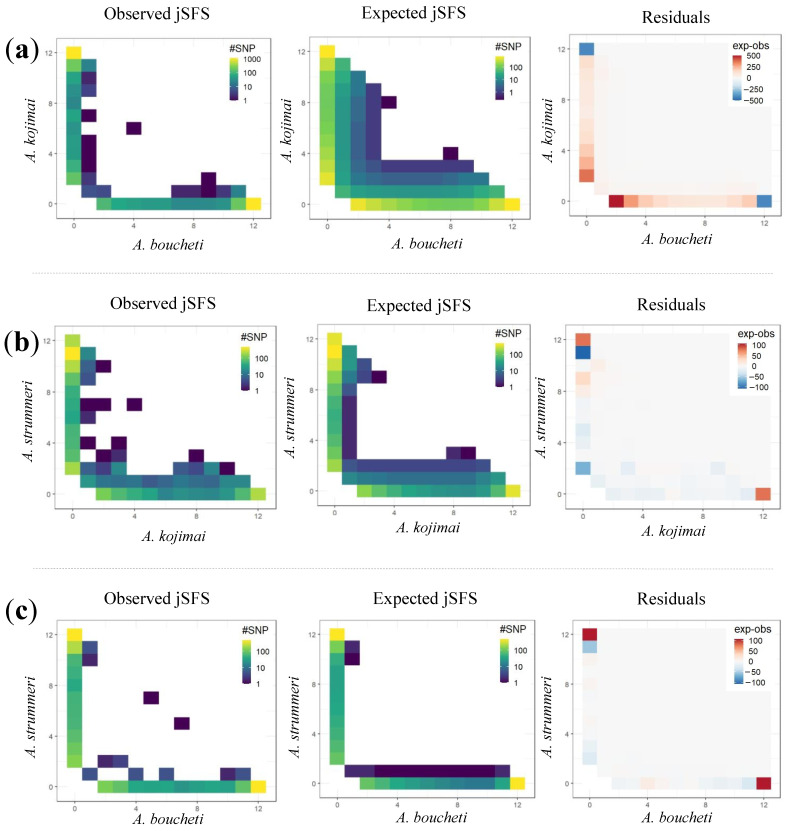
Comparison of the joint spectra of allele frequencies observed from the data (first column) and expected from the demographic model that provided the best fit in DILS (secondary contact with heterogeneous effective population size, second column) for the three pairs of species. The residuals (expected − observed) are displayed in the third column. (**a**) *A. boucheti*/*A. kojimai*; (**b**) *A. kojimai*/*A. strummeri*; (**c**) *A. boucheti*/*A. strummeri*. For the first pair of species (**a**), the position in the spectrum corresponds to the allele frequencies in *A. kojimai* (on the *y*-axis) and *A. boucheti* (on the *x*-axis), and the colour scale represents the number of SNPs with these allele frequencies. The white squares show an absence of SNPs having these allelic frequencies.

Using DILS to test whether admixtures may be the result of incomplete lineage sorting or introgression, we found that the secondary contact model (SC) fitted the observed data better than any other model in all ten runs for *A. boucheti*/*A. kojimai* and *A. kojimai*/*A. strummeri* (see the probabilities in Table S3 and joint site frequency spectrum in Figure 8). For the *A. boucheti*/*A. strummeri* species pair, SC was selected in seven runs, while the three remaining analyses better refer to ancient migration (AM). Since DILS is thought to be more efficient at distinguishing between these two broad scenarios than identifying further details [56], we did not further interpret the results for this last species pair (although we note that goodness of fit was highest for an SC model with heterogeneous *N*_e_ and *me*, Table S3). All our species pair simulations were also performed using a bottlenecked model of populations where the two sister populations were subjected to a size reduction after the ancestral population split. Introducing size reduction indeed clearly improved the goodness of fit of all the most sophisticated models implementing linked selection (2N) and semipermeable barriers to gene flow (2m). 

For the two other species pairs (*A. boucheti*/*A. kojimai*; *A. kojimai*/*A. strummeri*), increasing the complexity of the SC model by adding a heterogeneous effective population size (2N) among loci improved the fit between simulations and observed data (in all but one run, Tables S3 and S4), suggesting that a non-negligible fraction of loci are affected by linked selection. Variation in migration rates among loci across the genome (2m) was supported seven times out of 10 for each species pair (Table S3) but not in the analysis yielding the best fit for both species pairs (Table S3). This may be because the portion of the genome still permeable to inter-specific gene flow was probably very small between each pair of species.

**Figure 9:**
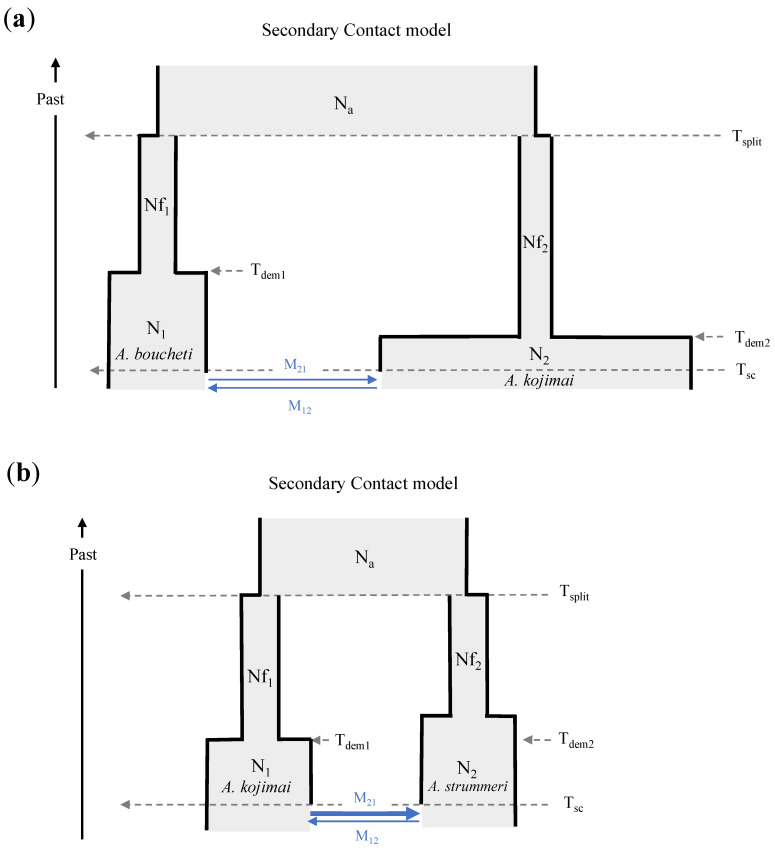
Demographic models that best explained the data observed in this study. N_1_ and N_2_: effective size of population 1 and 2; N_a_: effective size of the ancestral population; Nf_1_ and Nf_2_: effective size of population 1 and 2 after the split (differences in population size parameters are approximately reflected by the width of each box); T_split_: time of split at which the ancestral population subdivides in two populations; T_dem_: time of the expansion of the population; T_sc_: time of secondary contact at which the two populations start exchanging genes; M_12_ and M_21_: introgression rates from population 2 to 1 and from population 1 to 2, respectively (blue arrows). (**a**) Analysis for *A. boucheti*/*A. kojimai*; (**b**) analysis for *A. kojimai*/*A. strummeri*.

The estimated demographic parameters of the best models (using the random forest prediction method) are summarised in Table S4, and a graphical representation of the models with the best goodness of fit is presented in Figure 9. Note that most parameter estimates depend on mutation rate, which was set to µ = 10^−8^ as suggested by Wares and Cunningham [66] in *Littorina obtusata*, but the uncertainty of this parameter is large [67]. The effective size of the ancestral population was always higher than the effective sizes of the populations of the two daughter species after the split (Nf_1_ and Nf_2_ at T_split_; i.e., strong population bottlenecks). The size of daughter populations was then predicted to have increased at T_dem_ (time of expansion of the populations in this study) to reach current population sizes N_1_ and N_2_. Quantitative estimates of the timing of demographic change and population sizes varied strongly between runs and, thus, were quite imprecise. In most simulations, the sizes of the current populations remained, however, much lower than that of the ancestral population (Table S4). This finding may suggest that species isolation was also linked with a reduction of species range to geographic isolates, or that the ancestral population size between pairs of species was overestimated due to the lack of intermediate species such as *A. hessleri.*

Separation between the ancestral population and the two daughter populations was old (for analyses yielding the best fit, T_split_: around 141,340 and 133,200 generations for *A. boucheti*/*A. kojimai* and *A. kojimai*/*A. strummeri*, respectively) and preceded the period of population expansion (T_dem1_: 35,140/T_dem2_: 10,200 and T_dem1_: 25,740/T_dem2_: 55,500 generations for *A. boucheti*/*A. kojimai* and *A. kojimai*/*A. strummeri*, respectively). Then, a period of recovery of gene flow occurred between the populations (T_sc_: about 6900 and 12,000 generations for *A. boucheti*/*A. kojimai* and *A. kojimai*/*A. strummeri*, respectively). For each pair of species, the T_sc_/T_split_ ratio was of the same order of magnitude, indicating that the recovery of gene flow was very recent as compared to the formation of the species.

Despite an overall identical demographic pattern between the two pairs of species, there were slight differences (Figure 9). The ancestral effective size (N_a_), as well as the post-split effective sizes (Nf_1_ and Nf_2_) in the *A. kojimai*/*A. strummeri* pair, was much lower than in *A. boucheti*/*A. kojimai* (about half as much). Furthermore, although the T_split_ period was almost identical, the expansion of daughter population sizes was older in *A. boucheti*/*A. kojimai* to nearly reach contemporary effective sizes (N_1_/N_2_). Since *A. kojimai* was present in both pairs of species analysed, its current effective size could be estimated at about 130,000 individuals, while the other two species had lower current effective sizes (about 52,500 individuals for *A. boucheti* and 57,115 individuals for *A. strummeri*). One of the main differences between the species pairs was the rate of migrants (*4.Ne.m*) exchanged between the species. Indeed, in the *A. boucheti*/*A. kojimai* split, this rate seemed to be low and bidirectional, while, for the *A. kojimai*/*A. strummeri* split, the migration was strongly directed from *A. kojimai* to *A. strummeri* (Table S4). 

### 3.5. Divergences in Coding Sequences

The concatenated coding sequence alignment of the three species’ sets of transcripts corresponded to an overall alignment of 1,172,052 homologous nucleotide sites consisting of 1705 partial CDSs in the right coding frame, with 809,317 conserved and 362,735 informative sites. The observed net divergence (*d_A_*) between *Alviniconcha* species is of the same order of magnitude as those obtained with the ddRAD analyses: 1.6% between *A. kojimai* and *A. strummeri*, and around 2.8% between the two other species pairs (Table 2). The ratio of non-synonymous to synonymous substitutions (*d*_N_/*d*_S_), estimated using the free-ratio model M1 of CodeML implemented in AdaptSearch and a mid-rooted three-species tree, was equal to 0.124 between *A. kojimai* and *A. strummeri* and 0.133 between the two other species pairs (Table 2). Although non-synonymous and synonymous divergences were almost identical for the species pairs *A. boucheti*/*A. kojimai* (*d*_N_ = 0.0129, *d*_S_ = 0.0972) and *A. boucheti*/*A. strummeri* (*d*_N_ = 0.0128, *d*_S_ = 0.0960) and about 1.6 times greater than the pair *A. strummeri*/*A. kojimai*, the branch leading to *A. boucheti* accumulated non-synonymous changes a little bit more than the two other *Alviniconcha* branches (*d*_N_/*d*_S_ = 0.166 vs. *d*_N_/*d*_S_ = 0.150).

## 4. Discussion

### 4.1. Hydrothermal Vents in the Western Pacific Are Home to Three Sympatric Alviniconcha Species

Hydrothermal vent gastropods of the genus *Alviniconcha* have been found in the Indian and Western Pacific oceans, where a total of six species have been described [13,14]. Although species were delineated using up to three mitochondrial and seven nuclear genes [13,14,30], the most discriminating genetic marker was the mitochondrial *Cox1* gene [13,14]. Focusing on the three species *A. boucheti*, *A. kojimai*, and *A. strummeri*, which inhabit the Western Pacific, we found that the genetic divergence measured across the genome was consistent with the *Cox1* mtDNA results used previously to delineate species. Reference transcriptome assemblies counting nearly 1.2 million homologous sites across the three species and a RAD-seq dataset composed of over 4380 RAD loci sequenced in 36 to 250 individuals per species showed that the genetic divergence (measured as *d*_XY_) was ca. 2% between *A. kojimai* and *A. strummeri* and ca. 3% between *A. boucheti* and the two other species (Table 2). These values are consistent with the levels of genetic divergence generally observed between reproductively isolated species [27]. The SNP dataset (over 60,000 SNPs) derived from homologous RAD sequences also indicated that the genetic differentiation between species was strong (over 69% of the genetic variance explained by the first two principal components, Figure 6, and pairwise *F*_ST_ values between 0.84 and 0.92 from shared polymorphic loci, Table 2, while *F*_ST_ across basins within each species was between 0.003 and 0.02). Together with the mtDNA network and divergence estimates we obtained for the same set of individuals, all our results support the previous conclusions of Johnson et al. [13], Beinart et al. [65], and Breusing et al. [14] that *A. boucheti*, *A. kojimai*, and *A. strummeri* are genetically clearly distinct and probably at the end of their allelic lineage sorting.

While early observations showed that individuals of different *Alviniconcha* species had very similar external morphologies, here, we found that genetic divergence between species is accompanied by phenotypic differences. Cryptic species are common at hydrothermal vents [68,69], and *Alviniconcha* species have long been considered as such [13,29,70]. Yet, our morphological analyses across the distribution range of the three species confirm and extend the recent findings of Laming et al. [28] who described diagnostic morphological traits for these species in the hydrothermal fields of the Futuna Arc. Laming et al. [28] showed that they differed in the shape of the columella, radula, and snout, as well as in the arrangement of shell bristles. Building on their work on bristle arrangement, we confirm that individuals of *A. strummeri* in the Futuna Arc and North-Fiji and Lau Basins all had a regular arrangement of bristles of identical length. In addition, we observed that the bristle arrangements for the other two species were more variable, but with only two common types (type “bcd” and “d” in Figure 2). We also found that the specific “double-twisted” columella (or columellar fold) described by Laming et al. [28] for *A. kojimai* and *A. strummeri* in the Futuna Arc is a criterion that extends throughout the species’ ranges. It is, therefore, possible to identify *Alviniconcha* species from the Western Pacific by combining observations of bristle arrangement and columella shape. These observations can be made on board without dissection. In contrast, external shell measurements appear to be poorly discriminant (Figures S3 and S5).

Our study brings new information regarding the distribution of *Alviniconcha* in the Western Pacific. As recently reported, *A. kojimai* was present in the Futuna Arc, and Lau and North Fiji Basins [13,28], as well as at the entrance to the Manus Basin on Susu Volcanoes and the Woodlark Basin (Figure 1). It was absent from the Pacmanus area (Pual Ridge) of the Manus Basin, where *A. boucheti* was the only species found. *A. boucheti* was found in the Lau and Manus Basins and near the Futuna Volcanic Arc (Fati-Ufu and Mangatolo fields). In contrast to the previous study by Johnson et al. [13], we did not find this species at the White Lady/Ivory Tower sites in the North Fiji Basin (both of which are now extinct).However, noting its sporadic presence in this basin from previous reports is important to understand where the different species might have met contemporaneously (see Divergence History section below). It also highlights the speed at which species distribution can change (at least locally) and, thus, the difficulty in interpreting the overlap of observed ranges at a given time.

*A. kojimai* and *A. boucheti*, thus, have largely overlapping distributions, with *A. kojimai* being most abundant in the study area (where it represented 72% of the individuals we sampled) except in the Manus Basin (where *A. boucheti* represented 71% of the sampled individuals). Interestingly, this large-scale overlap in the species distribution range reflects local coexistence at finer spatial scales. These two species were often found in syntopy (i.e., a mixture of individuals within the same site, and even in a given patch). For instance, we found two samples with the two species in the Lau Basin, one in the Manus Basin (Susu) and one at a Futuna site. Both species were also found in syntopy at the base of hydrothermal vent chimneys at 3388 m depth at the newly discovered site “La Scala” in the Woodlark Basin (Boulart et al. [71]). On the basis of these observations, these two species seem to follow a longitudinal gradient where *A. boucheti* is currently expanding from the west to the east, while *A. kojimai* is expanding in the opposite direction. Although mixed with *A. kojimai* at some locations, *A. boucheti* was preferentially found (in 60% of the samples) on the walls of active hydrothermal chimneys as previously suggested in Beinart et al. [65].

The distribution of *A. strummeri* appeared more restricted. It occurred at a relatively low abundance at several diffuse vent sites of the southernmost area of the Lau Basin (Tow Cam, Tu’i Malila and ABE), as well as, although in lower abundance, on venting sites in the Futuna volcanic Arc and North Fiji Basin (Figure 1 and Table S1). When present, *A. strummeri* was always found in syntopy with at least one of the two other species (mostly *A. kojimai*). However, in situ observations suggest that it may be more peripheral to vent emissions and, thus, perhaps less well sampled than the other two species.

This study confirms that the three studied species of *Alviniconcha* gastropods coexist at small spatial scales in the Western Pacific (all three species were even found to occur simultaneously at the ABE vent site in the Lau Basin). In our collection boxes, 42% of samples contained at least two species (i.e., a mixing of individuals within a few tens of cm^2^). At the scale of a vent site, this proportion reached 60% (i.e., nine out of 15 sampled sites). This species coexistence in sympatry and even in syntopy raises interesting questions about the ecological preferences of these species and the role of geography and habitat in their evolution. To shed light on the biogeographic history of these gastropods, we conducted further analysis of their genomic divergence and polymorphism. 

### 4.2. A Long History of Divergence in Allopatry

All genomic datasets (mtDNA *Cox1*, transcriptomes, and nuclear RAD-seq) indicated that the divergence (measured either as *d*_XY_ or *d*_A_, Table 2) between *A. boucheti* and the ancestor of the two other species was ca. 1.4 to 1.6 times greater than the divergence between *A. kojimai* and *A. strummeri*. This was further confirmed by the fixation rates of non-synonymous (*d*_N_) and synonymous mutations (*d*_S_) in the three pairs of species, both of which indicate that *A. boucheti* was ca. 1.6 times more divergent than the two other species in the two coding compartments (mtDNA *Cox1* and transcriptomes). The older separation of *A. boucheti* from the two other species is also supported by the fact that it differs from its two congenerics by a different shell columella shape and a distinct endosymbiosis with *Campylobacteria* [14,65,72]. The almost equal and high divergence between *A. boucheti* and the two other species in all genomic compartments suggests that mutations were accumulated at the same rate between the two other species or that the demography of *A. kojimai* and *A. strummeri* was very similar. These results, therefore, suggest that the genetic divergence between species mostly reflects the time elapsed since speciation events. This finding is important because it is a critical assumption when using fossil-calibrated phylogenies for dating lineage splitting, as recently performed by Breusing et al. [14].

With mutations accumulating globally at a constant rate along lineages, ratios of divergence between species (e.g., *d*_A,sp1_/*d*_A,sp2_) appear relatively constant when measured from distinct genomic datasets, as explained above. By contrast, point divergence values (e.g., *d*_A,mtDNA_ and *d*_A,nuDNA_) are expected to differ due to differences in how these genomic regions are affected by evolutionary forces. Here, we found that mtDNA divergence was ca. 3.8 to 4.8 times greater than nuDNA divergence (see *d*_A_ and *d*_XY_ values in Table 2). It is unlikely that this discrepancy is due to the fourfold difference in effective population size *N* between mitochondrial and nuclear loci, because intra-specific diversity (reported as *π*) was low compared to the divergence. This means that *d*_A_ and *d*_XY_ are nearly equivalent, and the time since species split (>25 Ma according to Breusing et al. [14]) has long past the time needed for allelic lineage sorting between the three species (~8 Ne). Rather, our results are consistent with the neutral ratio of mitochondrial to nuclear mutation rate observed in non-vertebrates (typically around 5 [17]), although this ratio varies widely in molluscs (from 1.4 in *Fissurelloidea* to 91.9 in *Cristataria* [73], see also Allio et al. [74]). In the absence of strongly biased sex ratios, the contrast in evolutionary rate between mitochondrial and nuclear genomes could also be explained by differences in the strength of background selection. Here, according to *d*_N_/*d*_S_ values averaged over the three species, the strength of background selection was 16 times greater in the mitochondrial genome when compared with the nuclear one. This fits well with the previous hypothesis by Havird and Sloan [17] that a higher mitochondrial mutation rate could generate more positive selection for compensatory changes in nuclear genes interacting with the mitochondrial genome and/or that selective sweeps are more frequent in the mitochondrial genome while promoting divergence in the latter compartment.

Unexpectedly, the divergence measured from coding vs. noncoding nuclear regions did not differ much (transcriptomes vs. RAD sequences, Table 2). Purifying selection acting on the 3D structure and function of the encoded proteome instead predicts a lower divergence in coding regions [8]. Our results, therefore, suggest that loci were not chosen randomly along the genome but rather in well-conserved areas probably associated with coding regions. This is possible since we retained only the RAD sequences that were shared between the three species (which represented 10% of all RAD sequences, while 70% were amplified in a single species). Diversity and divergence estimated from RAD data may, therefore, be biased downwards (a caveat that cannot be avoided when looking at strongly divergent species by selecting a subset of their genomes).

Regarding the effect of selection on transcriptomes, we found that the non-synonymous fraction of the species divergence represented about 13% of the total observed divergence. This value is similar to those found in some marine species (0.10–0.20 [75]) and slightly lower than those obtained for mammals (0.20–0.25 [76,77]). The non-synonymous divergence for hydrothermal *Alviniconcha* species is, however, much higher than that found in hydrothermal alvinellids (0.01–0.04 [8]). These thermophilic worms indeed seem to experience stronger purifying selection due to functional constraints associated with the thermal denaturation of their proteins [8]. This result demonstrates that very low *d*_N_/*d*_S_ ratios (like in alvinellids) are not representative of all vent taxa, and that species experiencing less thermal constraints such as *Alviniconcha* [11,12] exhibit *d*_N_/*d*_S_ ratios similar to other marine species. In future studies, it will be interesting to look at the *d*_N_/*d*_S_ ratio between species for each gene to check for traces of disruptive (habitat) selection.

The analysis of genetic divergence between our three *Alviniconcha* species yielded one final intriguing observation. The mtDNA/nuDNA divergence ratio between *A. kojimai* and *A. strummeri* (*d*_A,mtDNA,strummeri_/*d*_A,nuDNA,kojimai_ ≈ 4.6) seemed slightly greater than between the two other species pairs (ca. 3.9). Although we cannot rule out the possibility that the strength of selection could have been greater on the mitochondrial genome of *A. kojimai* and *A. strummeri* after their separation, another hypothesis is that the nuclear genome of these two species experienced allelic rejuvenation through introgressive hybridisation. Although the genetic differentiation found between *A. kojimai* and *A. strummeri* on nuDNA was strong (*F*_ST_ = 0.84), it was not nearly fixed as found for the two other pairs (*F*_ST_ = 0.92 in both cases, Table 2). This is in line with the higher level of shared polymorphism between these species: over 3.5% of the SNPs genotyped in all three species were polymorphic in both *A. kojimai* and *A. strummeri*, while this figure dropped below 2% in the two other pairs. This is also clearly visible in the pairwise *F*_ST_ distribution (Figure S9) which shows a high number of *F*_ST_ values close to 0. These observations suggest that *A. kojimai* and *A. strummeri* did not reach reciprocal monophyly despite their high level of divergence on nuclear and mitochondrial genomes, which would be compatible with low levels of inter-specific gene flow. We used ancestry analyses and demographic inferences to explore this hypothesis among other historical scenarios of divergence for the three *Alviniconcha* species.

### 4.3. Historical Scenarios of Divergence

Results from ancestry analyses are compatible with the hypothesis of gene flow between the three *Alviniconcha* species, with traces of hetero-specific ancestry reaching up to 15% in some individuals (Figure 7). The alternative hypothesis of incomplete lineage sorting is unlikely given the high levels of divergence found in the mitochondrial compartment and the fact that most individuals displayed different levels of mixed ancestry depending on geography and the level of species mixing at relevant locations: traces of admixture in all species were mostly located in the Lau Basin and Futuna Arc, where the level of species mixture is also highest.

Demographic inferences performed with DILS supported the same conclusion: 27 of 30 runs (including models with the best fit) found that heterospecific gene flow occurred relatively recently after a long period of isolation between species. With this method, it is, however, difficult to date speciation events and secondary contacts in terms of number of generations, as it depends directly on an unknown mutation rate that could range from 5 × 10^−10^ to 3 × 10^−8^ [78,79]. With this range of mutation rates, the divergence time between *A. kojimai* and *A. strummeri* (estimated from the model with the best support) would be anywhere between 45,000 and 2.7 million generations. By contrast, using a clock calibration based on fossil records, Breusing et al. [14] estimated this split at about 25 Ma from their phylogenetic inferences. The same comparison for the *A. boucheti*/*A. kojimai* split leads to 47,113–2.8 million generations (DILS) vs. 48 Ma (using the same phylogenetic tree [14]). These values must be considered with caution as they are difficult to reconcile. Dating the separation of *A. kojimai* and *A. strummeri* to 2.7 millions generations on one hand and 25 Ma on the other hand leads to an approximate generation time of ca. 9 years, but all other estimates assuming a higher mutation rate lead to higher, unrealistic, generation times for vent species with high growth rate and early sexual maturation [80]. As a consequence, reconciling these analyses would probably lead to either a nuclear mutation rate of vent species by analogy to what was previously proposed for the mitochondrial *Cox1* gene [13,81,82] or a revision of these geotectonic- and/or fossil-driven molecular calibration dates.

One hypothetical scenario is that the collision of the Ontong-Java plateau with the Melanesian Arc about 18 Ma ago disrupted gene flow along the formerly well-connected South Fiji and Solomon ridges, thereby promoting allopatric diversification of the vent fauna in this region [83]. This collision led to the simultaneous rotation of the Vanuatu Arc and the Fiji Islands triggering the opening of (1) the North Fiji proto Basin about 10 Ma ago [84], (2) the Woodlark Basin (one of the oldest basins in the western Pacific) about 6 Ma ago as a result of continental rifting (i.e., the thinning of a tectonic plate due to stretching forces creating a volcanic zone [84,85]), and (3) the Manus Basin 3.5 to 4 Ma ago [86]. Finally, the expansion of the seafloor in the centre of the North Fiji proto Basin led to the opening of several ridge systems from which the Lau Basin was the most recently formed while expanding to the south, about 1 or 2 Ma ago [84]. Given that all our estimations converge towards secondary contact representing ca. 5% of the time since populations split (an estimate that is independent from mutation rates), it is possible that secondary contact between the three species happened during the formation of the Lau Basin. 

Under this hypothesis, the newly opened Lau Basin could have been subsequently colonised by a series of different allopatric species coming from the older Manus, Woodlark, and North Fiji ridge systems. We can, therefore, hypothesise that the opening of the Lau Basin near the active zone of Futuna allowed the mixing and hybridisation of the three *Alviniconcha* species, starting 1.2 Ma ago but with different timings (secondary contacts being more recent between *A*. *boucheti* and the two other species than between *A. strummeri* and *A. kojimai*). This hypothesis also accounts for our observation that most introgressed individuals were found in the Lau Basin and Futuna volcanic Arc (Figure 7) where the three species sometimes co-occur. To this extent and because of their genetic proximity, we cannot rule out the possibility that some of the introgressed alleles found in both *A. kojimai* and *A. strummeri* may also come from older interactions with both *A. hessleri* and *A. adamantis*, situated further north in the Mariana Basin/Trench. Genetic exchanges between our three species, however, do not fit well with larval dispersal modelling [87,88] which rather predict that inter-basin exchanges are more likely occurring stepwise via the North Fiji Basin. This suggests that rare inter-specific exchanges are either much older than thought or not specifically performed where the introgressed migrants are recruiting.

We were not expecting inter-specific exchange between the three species given the high level of mitochondrial and nuclear divergences. Usually, nuclear divergences greater than 2–3% should limit introgression between previously separated species due to the accumulation of genetic incompatibilities [24]. On the basis of genetic and morphological differences, previous studies on *Alviniconcha* species suggested that they were reproductively isolated. However, these studies were based on a few mitochondrial and highly conserved nuclear genes [13,14]. This is not the first time that traces of introgression have been observed between genetically distant species [26,27,89]. In fact, hybridisation still occasionally occurs between *Ciona robusta* and *Ciona intestinalis* despite a high transcriptome synonymous sequence divergence (14% [27] and 12–14% of mitochondrial *Cox3-Nd1* divergence [89]). In gastropods and echinoderms, cases also exist [90,91], as in the pulmonate gastropod genus *Rhagada*, where two species diverging by 30.2% on the *Cox1* gene are still able to produce sterile F1 [91], or in the ophiuroid genus *Acrocnida*, where cryptic species diverging by 19% on the *Cox1* gene are still able to locally hybridise and introgress in very sheltered habitats [92].

The maintenance of distinct species against gene flow is possible when reproductive isolation barriers efficiently prevent genome remixing [16]. In *Alviniconcha*, pre-zygotic barriers are almost certainly a strong obstacle to gene exchange. While we showed here that the species are sometimes in contact, they are often found in different habitats (although at small spatial scales) because of the metabolic requirements of their symbiotic bacteria [65,72]. Here, we found *A. kojimai* and *A. strummeri* preferentially on diffuse venting zones while *A. boucheti* was more frequently found on the wall of hydrothermal chimneys. In addition, post-zygotic barriers due to a long history of divergence in allopatry such as maladaptation [93] and sterility or unviability of hybrids [94,95] are likely to play a role in maintaining divergence between species. These barriers remain to be investigated.

## 5. Conclusions

This study showed the co-occurrence of three divergent *Alviniconcha* species (*A. kojimai*, *A. boucheti*, and *A. strummeri*) across the five back-arc Basins of the Western Pacific Ocean. The number of accumulated non-synonymous and synonymous substitutions between *A. boucheti*/*A. kojimai* and *A. boucheti*/*A. strummeri* was nearly identical on all genomic datasets, suggesting that divergence is proportional to the time since species separation. However, admixture analyses and demographic inferences clearly supported a scenario in which the three species evolved without gene flow for a long period of time (different geographic origins), followed by a relatively recent secondary contact with resumption of gene flow despite the strong accumulated divergence between species. These secondary contacts could coincide with the opening of the Lau Basin from 1.2 Ma onwards. With more than 60% of substitutions fixed between species, genetic (i.e., post-zygotic) barriers to gene flow are likely to be an important factor in reproductive isolation. Lastly, this study confirms that the species of the genus *Alviniconcha* can be distinguished using morphological characters. 

## Figures and Tables

**Table 1:** Intra-specific genetic diversity of the three species for the three genomic datasets: *Cox1* mitochondrial gene, ddRAD-seq nuclear SNPs, and RNA-seq nuclear SNPs. *N*: sample size; *K*: number of polymorphic sites; *H*: number of haplotypes; *Hd*: haplotype diversity, and *π*: nucleotide diversity.

	*N*	*K*	*H*	*Hd*	*π*
Mitochondrial (*Cox1*; 599 bp; 722 ind)					
*A. kojimai*	454	89	102	0.8	0.003
*A. boucheti*	243	67	62	0.91	0.004
*A. strummeri*	25	31	19	0.96	0.008
Transcriptomes (RNAseq; 1,186,131 bp; 7 ind)					
*A. kojimai*	2	10,672	2	1	0.004
*A. boucheti*	3	10,851	3	1	0.003
*A. strummeri*	2	12,010	2	1	0.005
Genome (ddRAD-seq; 498 ind)					
*A. kojimai*	250	21,397			0.0013
*A. boucheti*	212	40,879			0.0014
*A. strummeri*	36	25,801			0.0014

**Table 2:** Inter-specific genetic divergence and differentiation for the three genetic datasets: *Cox1* mitochondrial gene (599 bp), ddRAD-seq SNPs (60,084 SNPs), and nuclear encoding RNAseq (1,186,131 bp). *d*_A_: net divergence; *d*_XY_: absolute divergence; *F*_ST_: genetic differentiation; *d*_N_: rate of non-synonymous substitutions; *d*_S_: rate of synonymous substitutions.

	*d*_A_	*d*_XY_	*F*_ST_	*d*_N_/*d*_S_	*d*_N_	*d*_S_
Mitochondrial (*Cox1*; 599 bp; 722 ind)						
*A. kojimai*/*A. boucheti*	0.123	0.126	0.974	0.006	0.005	0.784
*A. kojimai*/*A. strummeri*	0.086	0.091	0.961	0.015	0.007	0.468
*A. boucheti*/*A. strummeri*	0.118	0.124	0.967	0.003	0.002	0.793
Transcriptomes (RNAseq; 1,186,131 bp; 7 ind)						
*A. kojimai*/*A. boucheti*	0.028	0.031		0.133	0.013	0.097
*A. kojimai*/*A. strummeri*	0.016	0.020		0.124	0.008	0.062
*A. boucheti*/*A. strummeri*	0.027	0.031		0.134	0.013	0.096
Genome (ddRAD-seq; 60,084 SNPs; 498 ind)						
*A. kojimai*/*A. boucheti*	0.031	0.031	0.922			
*A. kojimai*/*A. strummeri*	0.018	0.018	0.842			
*A. boucheti*/*A. strummeri*	0.031	0.031	0.917			

## Data Availability

Individual fastq files and *Cox1* sequences are available from NCBI under accession number PRJNA768636.

## Supplementary Materials

The following supporting information can be downloaded at: https://www.mdpi.com/article/10.3390/genes13060985/s1, Figure S1: Shell traits measured with a calliper on *Alviniconcha* individuals during the Chubacarc expedition from Chiu et al. [40]. SL: total length, SW: total width, ei and fh: length and width of the operture, ce and bf: spires lengths; Figure S2: Number of conserved SNPs and error rate as a function of *n*, *m* and *M* in Stacks; Figure S3: Distribution of six morphometric variables measured in *Alviniconcha boucheti* (purple, *n* = 247 ind.), *A. kojimai* (turquoise, *n* = 409), and *A. strummeri* (orange, *n* = 44). SL: total length, SW: total width, ei and fh: length and width of the operture, ce and bf: spires lengths; Figure S4: Distribution of the total length (SL) as a function of sampling basin in *Alviniconcha boucheti* (purple, *n* = 247 ind.), *A. kojimai* (turquoise, *n* = 409), and *A. strummeri* (orange, *n* = 44); Figure S5: Principal component analysis of five transformed morphometric variables (see text) for *Alviniconcha boucheti* (purple, *n* = 247 ind.), *A. kojimai* (turquoise, *n* = 409), and *A. strummeri* (orange, *n* = 44). The first four components shown here explain 31.2%, 25.4%, 18.1%, and 16.5% of the variance, respectively. The second principal component seems to be linked mildly with species identity; Figure S6: Principal component analysis of five transformed morphometric variables (see text) for *Alviniconcha boucheti* (circle, *n* = 247 ind.), *A. kojimai* (triangle, *n* = 409), and *A. strummeri* (square, *n* = 44) according to the sampling basin (Fiji: green; Futuna: pink; Lau: yellow; Pacmanus: turquoise; Susu: blue and Woodlark: purple). The first four components shown here explain 31.2%, 25.4%, 18.1%, and 16.5% of the variance, respectively. The second principal component seems to be linked mildly with locality; Figure S7: Linear discriminant analysis of species identity based on five transformed morphometric variables (see main text) for *Alviniconcha boucheti* (purple, *n* = 247 ind.), *A. kojimai* (turquoise, *n* = 409), and *A. strummeri* (orange, *n* = 44). The combination of linear discriminants only allows a partial reclassification of species (75%); Figure S8: Haplotype network of 722 individuals of *Alviniconcha* spp for *Cox1* partial mitochondrial sequence rooted with *Ifremeria nautilei* (*n*= 4). Circles represent individual haplotypes, while circle size is proportional to haplotype frequency. The traits on the branches indicate the number of mutations between haplotypes. Orange: Lau Basin; Green: North-Fiji Basin, Pink: volcanic zone of Futuna, Yellow: Mangatolo site, Purple: Woodlark Basin, Blue: Manus Basin and Red: Marianna Trench; Figure S9: Distribution of *F*_ST_ values by species pair, (a) *A. kojimai*/*A. strummeri* on 39,533 SNP, (b) *A. boucheti*/*A. strummeri* on 45,514 SNP and (c) *A. kojimai*/*A. boucheti* on 50,093 SNP; Figure S10: Values of the cross-entropy criterion as a function of the number of factors used in snmf runs; Table S1: Specimens used for each genetic analysis. Details are also available from NCBI Biosamples SAMN22059315-SAMN22060075 and SAMN22155691-SAMN22155716; Table S2: Pairwise genetic differentiation (estimated by *F*_ST_) between populations of each species. (a) For *A. boucheti*, (b) For *A. strummeri* and (c) For *A. kojimai*; Table S3: Likelihood of demographic models in the hierarchical comparison of alternate models in DILS. The probability (P.) of ongoing migration is the likelihood of current migration vs. current isolation. P. SC gives the support for secondary contact against isolation with migration (within the context of ongoing migration). The probability (P.) of ongoing isolation is the likelihood of current isolation vs. current migration. P. AM gives support for ancient migration against strict isolation (within the context of ongoing isolation). The last four columns give the likelihood of heterogeneous/homogeneous effective population size and migration across loci along the genome; Table S4: Demographic parameters estimates under the Secondary Contact model. N_1_ and N_2_: effective size of population 1 and 2; N_a_: effective size of the ancestral population; Nf_1_ and Nf_2_: effective size of population 1 and 2 after the split calculated by N_a_ × founders1 or N_a_ × founders2; shape_N_1 and shape_N_2: shape parameter α (resp.β) of the Beta (α,β) distribution for N_e_; T_split_: time of split at which the ancestral population subdivides in two populations (in generations); T_dem_: time of the reduction of the effective size population; T_sc_: time of secondary contact at which the two populations start exchanging genes (in generations); M_12_ and M_21_: introgression rates from population 2 to 1 and from population 1 to 2, respectively (in number of migrants per generation); For each analysis performed, the index between brackets represents the species population.
