# Supplementary material for: Inter-Specific Genetic Exchange Despite Strong Divergence in Deep-Sea Hydrothermal Vent Gastropods of the Genus Alviniconcha
Source: Genes (Basel). 2022 May 31;13(6):985. doi: 10.3390/genes13060985 (PMC9223106; doi:10.3390/genes13060985)
Supplement: Supplementary file 1 [file genes-13-00985-s001.zip › genes-1614743-supplementary.pdf]

Supplementary Material:

Table S1. Specimens used for each genetic analysis. Details are also available from NCBI Biosamples SAMN22059315-SAMN22060075 and SAMN22155691-SAMN22155716.

Sample	Site	Field	Basin	Habitat	*A. kojimai*	*A. boucheti*	*A. strummeri*	Not identified	Longitude	Latitude	depth (m)	Date	Analysis
721-GBT1	Tow Cam		Lau	diffuse zone	33		1		176°08'15,4" W	20°19'04,4" S	2716	2019	COI/ddRAD/ morphology
721-GBT6	Tow Cam		Lau	diffuse zone	44	5			176°08'15,8" W	20°19'05,1" S	2711	2019	COI/ddRAD/ morphology
721-GBT7	Tow Cam		Lau	chimney		20			176°08'12,7" W	20°18'59,2" S	2714	2019	COI/ddRAD/ morphology
	Tow Cam		Lau	diffuse zone		2					2715	2009	COI/RNAseq
722-GBT7	Tui Malila		Lau	diffuse zone	31		5		176°34'04,2" W	21°59'15,2" S	1899	2019	COI/ddRAD/ morphology
722-GBT1	Tui Malila		Lau	diffuse zone	33		19	3	176°34'05,9" W	21°59'21,2" S	1886	2019	COI/ddRAD/ morphology
722-GBT5	Tui Malila		Lau	diffuse zone	32		15		176°34'05,5" W	21°59'21,4" S	1884	2019	COI/ddRAD/ morphology

	Tui Malila		Lau	diffuse zone	2		2				1885	2009	COI/RNAseq
731-GBT3	ABE		Lau	chimney	2	26	1	1	176°11'28,9" W	20°45'47,1" S	2149	2019	COI/ddRAD/ morphology
	ABE		Lau	diffuse zone		1					2145	2009	COI/RNAseq
726-GBT4	Mangatolo		Lau	diffuse zone		26			174°39'12,7" W	15°24'52,8" S	2031	2019	COI/ddRAD/ morphology
726-GBT3	Mangatolo		Lau	chimney		4			174°39'12,5" W	15°24'52,7" S	2031	2019	COI/ddRAD/ morphology
726-PBT6	Mangatolo		Lau	diffuse zone	18				174°39'19,9" W	15°24'57,7" S	2039	2019	COI/ddRAD/ morphology
726-GBT2	Mangatolo		Lau	diffuse zone	3				174°39'12,6" W	15°24'52,5" S	2031	2019	COI/ddRAD/ morphology
724-GBT4	Phoenix		North Fiji	diffuse zone	39				173°55'7,6" E	16°57'0,0" S	1961	2019	COI/ddRAD/ morphology
724-PBT4	Phoenix		North Fiji	diffuse zone	33		9		173°55'4,7" E	16°56'57,8" S	1973	2019	COI/ddRAD/ morphology
727-GBT2	AsterX	Fatu Kapa	Futuna	diffuse zone	26				177°09'07,9" W	14°45'06,5" S	1562	2019	COI/ddRAD/ morphology
727-GBT4	Stephanie	Fatu Kapa	Futuna	chimney	16		1		177°09'57,6" W	14°44'14,7" S	1547	2019	COI/ddRAD/ morphology

728-GBT2		Fati Ufu	Futuna	chimney	27		3	2	177°11'07,0" W	14°45'35,8" S	1519	2019	COI/ddRAD/ morphology
728-PBT4		Fati Ufu	Futuna	diffuse zone	40		1		177°11'04,9" W	14°45'35,3" S	1519	2019	COI/ddRAD/ morphology
728-GBT6		Fati Ufu	Futuna	chimney	3	4			177°11'05,9" W	14°45'35,2" S	1518	2019	COI/ddRAD/ morphology
733-GBT2	Big Papi	Pacmanus	Manus	chimney		24			151°40'20,1" E	3°43'43,9" S	1708	2019	COI/ddRAD/ morphology
733-GBT8	Fenway	Pacmanus	Manus	diffuse zone		24			151°40'22,4" E	3°43'41,2" S	1696	2019	COI/ddRAD/ morphology
733-GBT9	Solwara8	Pacmanus	Manus	chimney		13			151°40'27,5" E	3°43'49,3" S	1737	2019	COI/ddRAD/ morphology
733-PBT7	Solwara8	Pacmanus	Manus	chimney		5			151°40'26,6" E	3°43'50,1" S	1734	2019	COI/ddRAD/ morphology
734-GBT9		Pacmanus	Manus	chimney		28			152°6'2,8" E	3°43'17,2" S	1659	2019	COI/ddRAD/ morphology
736-GBT3	Solwara1	Susu	Manus	chimney	24				152°5'47,0" E	3°47'22,1" S	1505	2019	COI/ddRAD/ morphology
736-GBT10	North Su	Susu	Manus	diffuse zone	22				152°6'2,8" E	3°47'56,0" S	1218	2019	COI/ddRAD/ morphology
737-GBT10	South Su	Susu	Manus	diffuse zone		24			152°6'18,6" E	3°48'35,0" S	1353	2019	COI/ddRAD/ morphology

737-PBT5	South Su	Susu	Manus	chimney	17	13	152°6'17,5" E	3°48'29,8" S	1300	2019	COI/ddRAD/ morphology
737-GBT7	South Su	Susu	Manus	diffuse zone		24	152°6'17,9" E	3°48'31,8" S	1343	2019	COI/ddRAD/ morphology
738-GBT10	Scala		Woodla rk	chimney	24		155°03'09,6" E	9°47'56,7" S	3388	2019	COI/ddRAD/ morphology
739-GBT10	Scala		Woodla rk	chimney	24		155°03'07,0' E	9°47'56,3' S	3344	2019	COI/ddRAD/ morphology
739-PBT5	Scala		Woodla rk	chimney		24	155°03'08,1" E	9°47'56,0" S	3353	2019	COI/ddRAD/ morphology

Table S2. Pairwise genetic differentiation (estimated by F_{ST}) between populations of each species. a) For *A. boucheti*, b) For *A. strummeri* and c) For *A. kojimai*.

a)	Lau Basin	Futuna arc	Manus Basin
Lau basin	-	-	-
Futuna	0	-	-
Manus basin	0.002	0.003	-
Woodlark basin	0.003	0.003	0

b)	Lau Basin	Futuna arc
Lau basin	-	-
Futuna	0	-
Fiji basin	0.022	0.021

c)	Lau Basin	Futuna arc	Fiji Basin	Manus Basin
Lau basin	-	-	-	-
Futuna	0	-	-	-
Fiji basin	0.004	0.004	-	-
Manus basin	0.011	0.011	0.008	-
Woodlark basin	0.011	0.010	0.008	0

Table S3. Likelihood of demographic models in the hierarchical comparison of alternate models in DILS. The probability (P.) of ongoing migration is the likelihood of current migration vs. current isolation. P. SC gives the support for secondary contact against isolation with migration (within the context of ongoing migration). The probability (P.) of ongoing isolation is the likelihood of current isolation vs. current migration. P. AM gives support for ancient migration against strict isolation (within the context of ongoing isolation). The last four columns give the likelihood of heterogeneous/ homogeneous effective population size and migration across loci along the genome.

Species 1	Species 2	P. Ongoing migration	P. Ongoing isolation	P. SC	P. AM	P. N-heterogeneous	P. N-homogeneous	P. M-heterogeneous	P. M-homogeneous
A. boucheti	*A. kojimai*	0.93		0.95		0.65		0.42	
A. boucheti	*A. kojimai*	0.87		0.96			0.57	0.60	
A. boucheti	*A. kojimai*	0.95		0.95		0.56			0.51
A. boucheti	*A. kojimai*	0.94		0.96		0.59		0.46	
A. boucheti	*A. kojimai*	0.94		0.91		0.58		0.45	
A. boucheti	*A. kojimai*	0.96		0.95		0.65			0.54
A. boucheti	*A. kojimai*	0.87		0.95		0.63			0.60

A. boucheti	*A. kojimai*	0.91	0.89	0.67	0.51	
A. boucheti	*A. kojimai*	0.94	0.93	0.79	0.47	
A. boucheti	*A. kojimai*	0.90	0.90	0.65	0.48	
A. kojimai	*A. strummeri*	0.97	0.87	0.70	0.62	
A. kojimai	*A. strummeri*	0.97	0.80	0.67	0.59	
A. kojimai	*A. strummeri*	0.98	0.90	0.84	0.65	
A. kojimai	*A. strummeri*	0.98	0.89	0.81	0.55	
A. kojimai	*A. strummeri*	0.97	0.88	0.79	0.51	
A. kojimai	*A. strummeri*	0.98	0.90	0.72		0.60
A. kojimai	*A. strummeri*	0.97	0.88	0.71	0.64	
A. kojimai	*A. strummeri*	0.99	0.86	0.74	0.47	
A. kojimai	*A. strummeri*	0.98	0.78	0.77		0.52

A. kojimai	*A. strummeri*	0.97		0.88		0.71			0.59
A. boucheti	*A. strummeri*	0.90		0.92		0.66			0.47
A. boucheti	*A. strummeri*	0.88		0.93			0.74	0.61	
A. boucheti	*A. strummeri*		0.89		0.60	0.70			
A. boucheti	*A. strummeri*	0.90		0.88		0.63			0.49
A. boucheti	*A. strummeri*	0.87		0.87		0.65			0.39
A. boucheti	*A. strummeri*	0.89		0.81		0.68		0.68	
A. boucheti	*A. strummeri*	0.87		0.90		0.59		0.43	
A. boucheti	*A. strummeri*	0.90		0.82		0.80		0.56	
A. boucheti	*A. strummeri*		0.90		0.72	0.78			
A. boucheti	*A. strummeri*		0.92		0.65	0.81			

Table S4. Demographic parameters estimates under the Secondary Contact model. N_1 and N_2: effective size of population 1 and 2; N_a: effective size of the ancestral population; Nf_1 and Nf_2 : effective size of population 1 and 2 after the split calculated by N_a x founders1 or N_a x founders2 ; shape_N_1 and shape_N_2: shape parameter α (resp.β) of the Beta (α,β) distribution for N_e; T_{split}: time of split at which the ancestral population subdivides in two populations (in generations); T_{dem} : time of the reduction of the effective size population; T_{sc}: time of secondary contact at which the two populations start exchanging genes (in generations); M_{12} and M_{21}: introgression rates from population 2 to 1 and from population 1 to 2, respectively (in number of migrants per generation); For each analysis performed, the index between brackets represents the species population.

Species 1	Species 2	Euclidean distance	N_a	Nf_1	Nf_2	N_1	N_2	Shape_ N_1	Shape_ N_2	T_{split}	T_{dem1}	T_{dem2}	T_{sc}	T_{sc}/T_{split}	T_{am}	M_{12}	shape_ M12_a	shape_ M12_b	M_{21}	shape_ M21_a	shape_ M21_b
A. boucheti	*A. kojimai*	0.95	266 855	16 529	2 057	62 340	49 340	1.41	0.72	139 580	5 440	87 920	3 480	0.03		1.32	1.27	3.21	1.88	0.90	0.73
A. boucheti	*A. kojimai*	1.52	221 825	10 577	22 611	33 110	347 690	1.46	1.86	185 580	80 740	5 320	2 960	0.02		2.24	1.35	2.55	1.55	3.45	3.40
A. boucheti	*A. kojimai*	1.08	291 415	13 702	1 769	396 915	53 375			96 060	6 660	75 280	7 100	0.07		0.49	1.03	2.01	1.02	0.51	1.39
A. boucheti	*A. kojimai*	0.78	263 905	11 250	10 786	52 500	280 335	2.06	1.70	141 340	35 140	10 200	6 900	0.05		0.72			0.71		
A. boucheti	*A. kojimai*	0.99	262 445	7 797	20 072	38 640	298 155	2.37	2.21	154 160	41 980	7 820	10 040	0.07		0.72			0.42		

A. boucheti	*A. kojimai*	0.98	269 830	11 203	15 159	42 260	81 105	3.02	2.00	145 640	40 340	24 020	2 700	0.02	1.61	1.81	3.90	1.93	0.91	2.58
A. boucheti	*A. kojimai*	1.21	237 620	11 301	1 138	37 105	39 755	2.79	3.98	156 620	50 300	144 420	2 340	0.02	2.00	4.66	2.82	1.91	1.49	2.07
A. boucheti	*A. kojimai*	1.32	266 985	29 350	19 698	20 315	110 000	2.38	1.46	151 780	120 060	14 340	5 380	0.04	1.41	1.65	3.47	0.75	1.24	0.94
A. boucheti	*A. kojimai*	1.32	238 975	12 111	19 347	33 020	90 130	1.93	2.49	172 500	31 940	23 560	2 680	0.02	1.82	3.23	0.94	2.52	1.39	3.08
A. boucheti	*A. kojimai*	1.11	283 840	68 343	10 130	18 190	51 420	1.86	0.77	122 100	122 700	49 680	1 440	0.01	2.93			3.72		
A. kojimai	*A. strummeri*	0.78	130 795	11 040	4 816	103 615	61 535	1.55	2.74	126 720	22 620	69 980	10 320	0.08	0.36	1.62	1.89	4.18	4.04	2.14
A. kojimai	*A. strummeri*	0.94	145 945	14 254	10 947	112 745	59 170	3.40	2.37	102 560	18 020	42 540	7 300	0.07	0.34	2.76	1.25	4.94	4.56	1.60
A. kojimai	*A. strummeri*	0.92	115 855	14 612	4 124	52 910	46 635	1.49	3.21	143 220	35 460	103 940	7 940	0.06	0.48	4.39	1.15	5.15	3.28	2.79
A. kojimai	*A. strummeri*	0.76	114 260	7 936	21 847	64 615	169 210	1.28	2.35	143 180	60 080	8 860	8 540	0.06	0.68	0.82	2.94	4.72	4.10	0.20
A. kojimai	*A. strummeri*	1.03	118 035	5 361	10 031	83 595	108 870	1.61	4.05	106 720	33 340	36 180	5 700	0.05	1.22	2.92	0.96	5.57	5.05	1.00

A. kojimai	*A. strummeri*	0.78	127 500	11 676	12 719	53 885	67 510	2.25	4.69	125 280	49 580	42 680	8 480	0.07		0.48	0.76	1.50	4.74	4.27	3.60
A. kojimai	*A. strummeri*	0.75	109 300	12 043	12 553	64 980	74 600	1.40	1.95	136 620	37 640	35 720	8 240	0.06		0.33			4.62		
A. kojimai	*A. strummeri*	0.81	124 230	6 130	17 370	54 150	104 685	1.83	3.48	124 080	67 360	15 460	10 660	0.09		0.36	2.25	3.35	3.86	4.23	1.07
A. kojimai	*A. strummeri*	1.35	156 270	10 328	6 952	492 275	84 105	1.56	1.50	74 640	10 260	29 780	6 880	0.09		0.33			5.58		
A. kojimai	*A. strummeri*	0.72	131 210	12 654	7 038	82 960	57 115	1.55	4.70	133 200	25 740	55 500	12 000	0.09		0.37			4.08		
A. boucheti	*A. strummeri*	1.31	195 030	13 428	30 349	36 935	32 095	3.45	0.26	158 020	38 220	62 500			82 760	1.91	2.55	5.12	3.14	3.17	2.91
A. boucheti	*A. strummeri*	1.16	157 765	10 731	12 132	54 930	25 145	1.73	3.17	113 160	24 360	39 520	2 060	0.02		1.24			3.75		
A. boucheti	*A. strummeri*	1.53	182 395	16 649	21 539	546 525	1 010 655	4.48	2.27	140 740	7 660	20			63 980	2.11			3.33		
A. boucheti	*A. strummeri*	1.21	151 795	10 821	15 923	46 850	26 230	2.37	1.27	112 820	23 820	41 560	2 960	0.03		0.56	2.72	1.04	2.63	2.33	0.98

A. boucheti	*A. strummeri*	1.29	181 940	10 338	69 598	44 435	16 660			70 920	15 720	73 960	2 260	0.03		0.93	1.59	2.52	2.11	1.75	1.85
A. boucheti	*A. strummeri*	1.23	231 740	17 420	18 963	27 395	28 245	4.22	2.16	232 520	62 080	88 860			104 420	4.14			3.11		
A. boucheti	*A. strummeri*	1.01	167 975	1 925	12 828	36 065	22 980	1.65	0.67	91 580	74 900	47 720	2 320	0.03		0.68			2.77		
A. boucheti	*A. strummeri*	1.01	163 150	2 842	30 770	39 650	15 625	2.14	3.88	107 360	85 360	104 780	2 760	0.03		0.50	4.66	3.89	2.44	1.16	1.53
A. boucheti	*A. strummeri*	1.41	145 360	14 251	22 573	34 770	26 695	3.15	2.77	139 300	49 780	3 220	6 120	0.04		0.39			1.27		
A. boucheti	*A. strummeri*	1.26	167 780	17 548	21 056	83 455	21 185	2.84	1.93	108 140	360	5 760	3 200	0.03		0.72	3.67	1.92	2.19	4.01	4.51

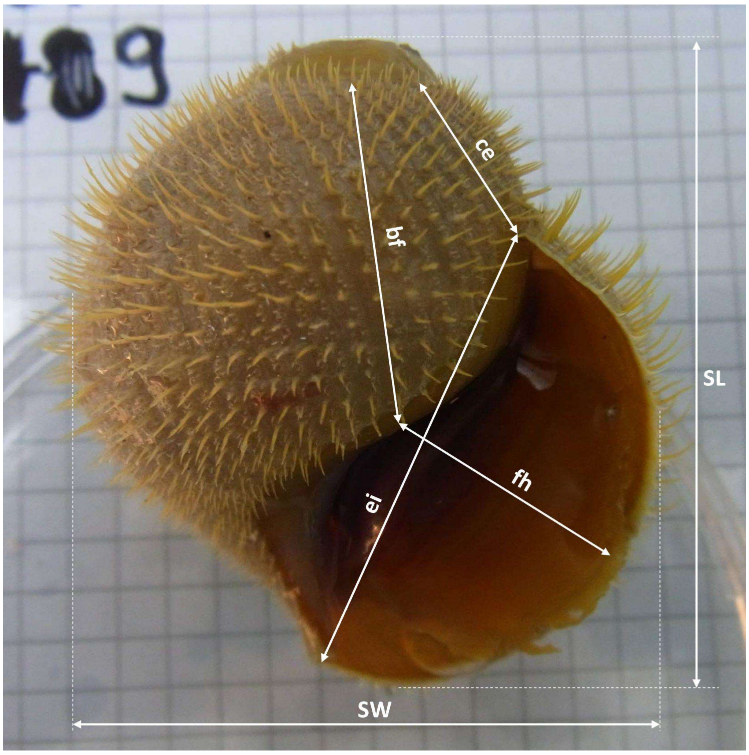

Figure S1. Shell traits measured with a calliper on *Alviniconcha* individuals during the Chubacarc expedition from Chiu et al. [40]. SL: total length, SW: total width, ei and fh: length and width of the operture, ce and bf: spires lengths.

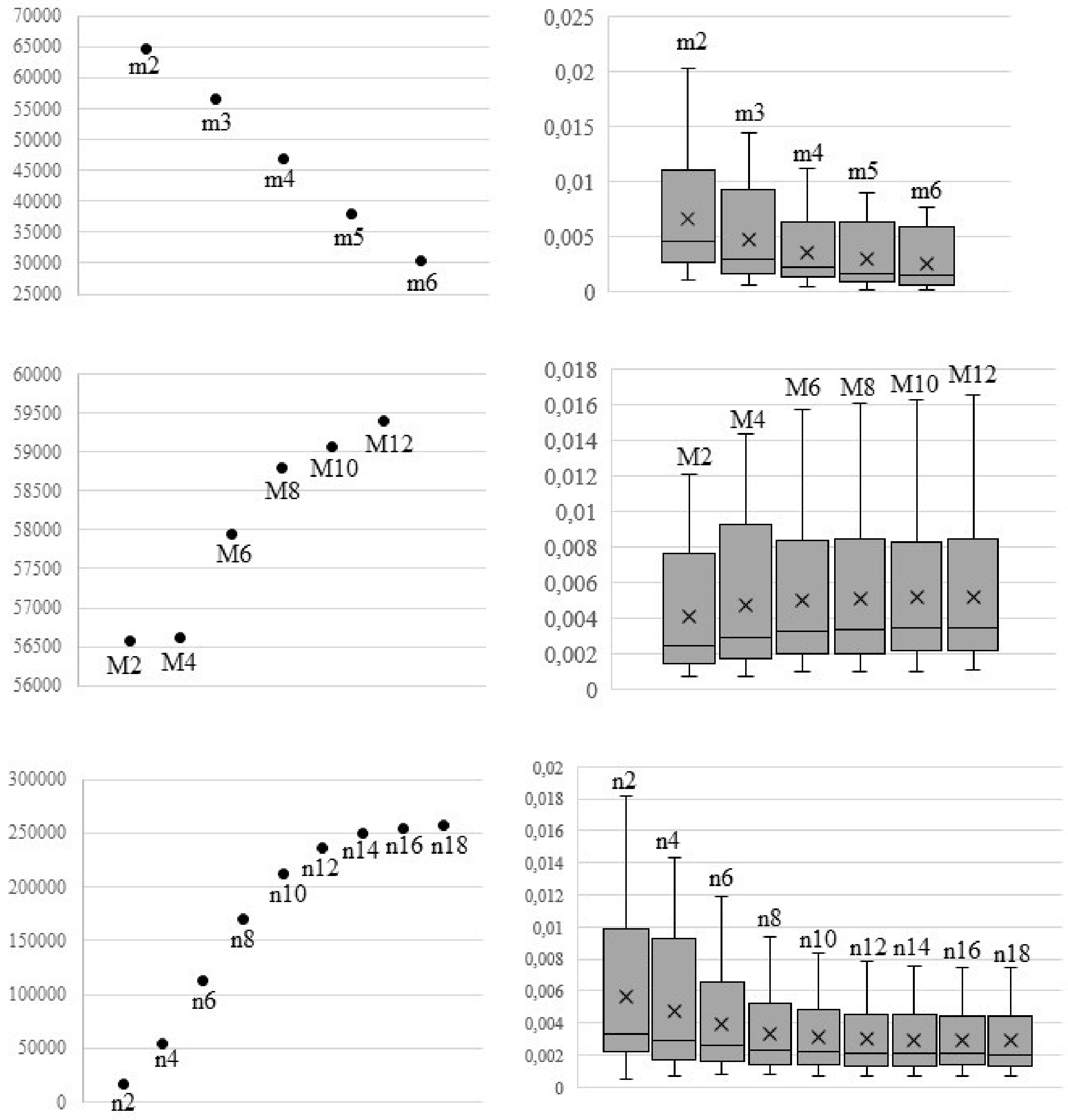

Figure S2. Number of conserved SNPs and error rate as a function of *n*, *m* and *M* in Stacks.

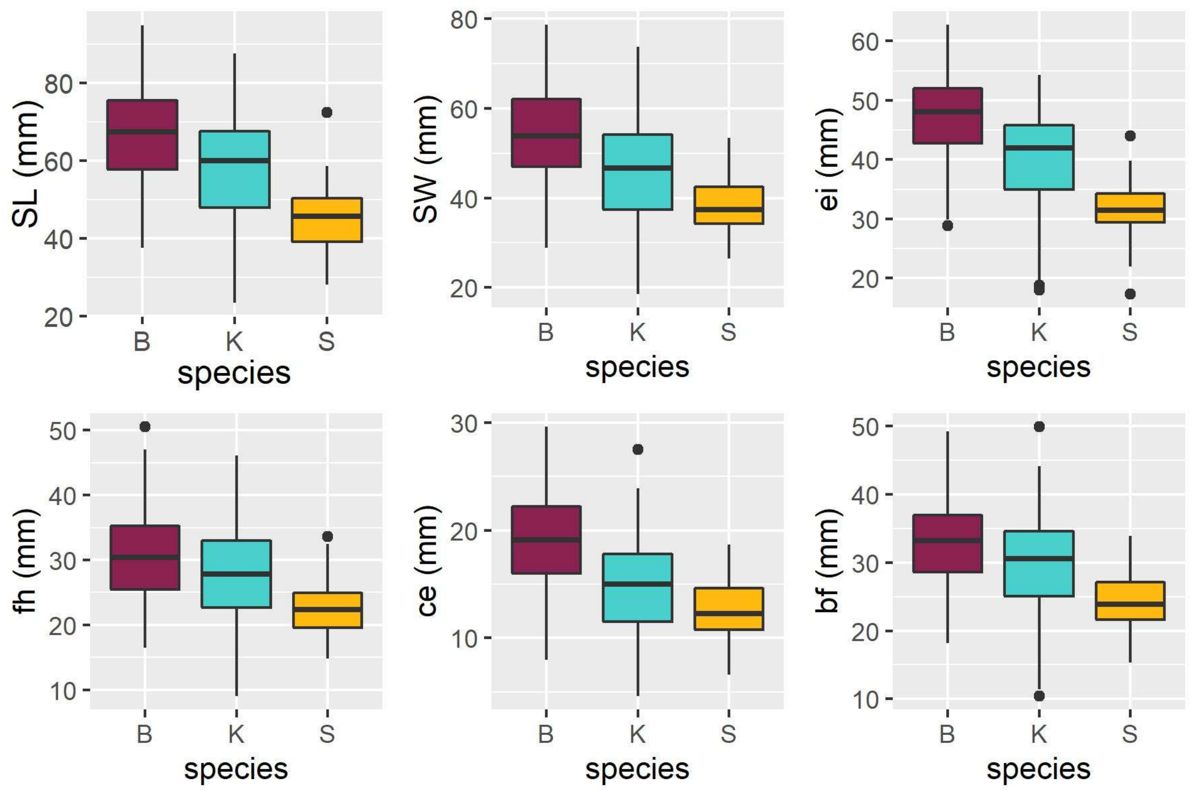

Figure S3. Distribution of six morphometric variables measured in *Alviniconcha boucheti* (purple, *n*=247 ind.), *A. kojimai* (turquoise, *n*=409), and *A. strummeri* (orange, *n*=44). SL: total length, SW: total width, ei and fh: length and width of the operture, ce and bf: spires lengths.

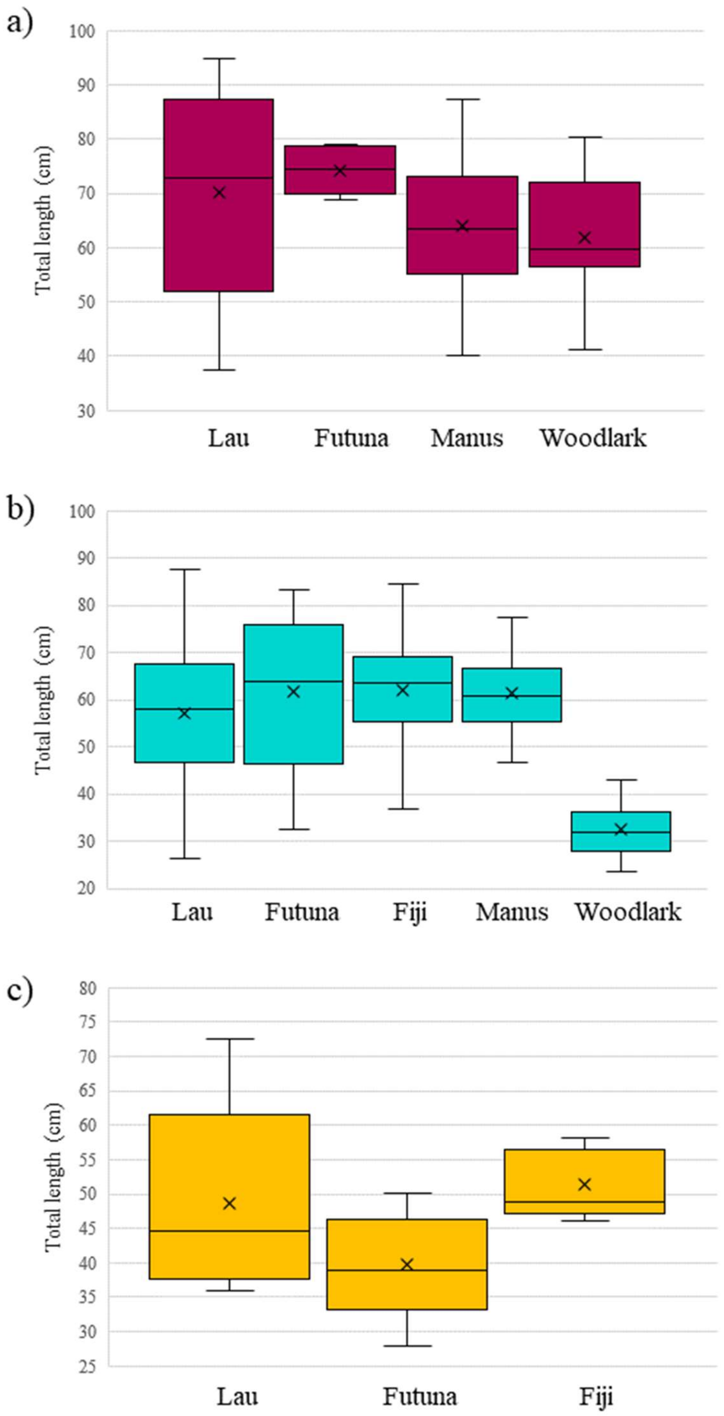

Figure S4. Distribution of the total length (SL) as a function of sampling basin in *Alviniconcha boucheti* (purple, *n*=247 ind.), *A. kojimai* (turquoise, *n*=409), and *A. strummeri* (orange, *n*=44).

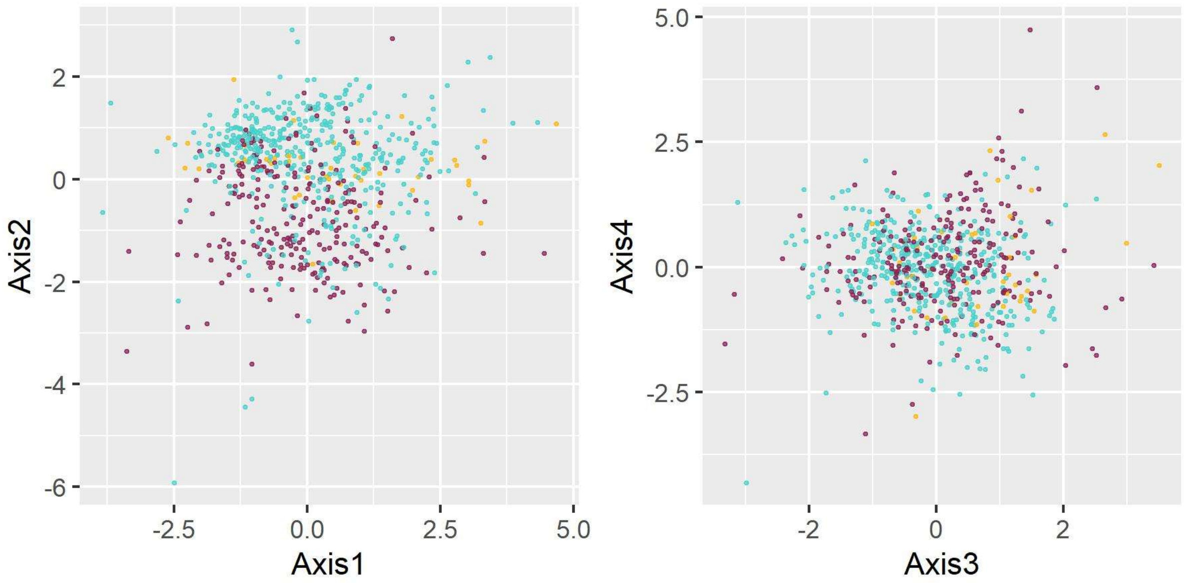

Figure S5. Principal component analysis of five transformed morphometric variables (see text) for *Alviniconcha boucheti* (purple, *n*=247 ind.), *A. kojimai* (turquoise, *n*=409), and *A. strummeri* (orange, *n*=44). The first four components shown here explain 31.2%, 25.4%, 18.1%, and 16.5% of the variance, respectively. The second principal component seems to be linked mildly with species identity.

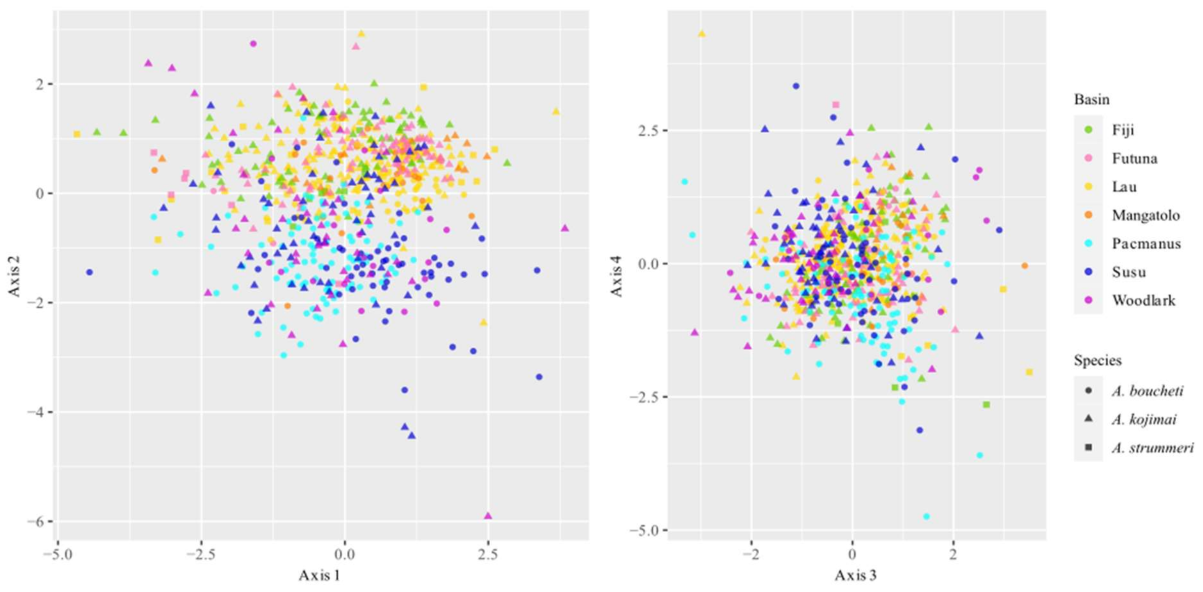

Figure S6. Principal component analysis of five transformed morphometric variables (see text) for *Alviniconcha boucheti* (circle, *n*=247 ind.), *A. kojimai* (triangle, *n*=409), and *A. strummeri* (square, *n*=44) according to the sampling basin (Fiji: green; Futuna: pink; Lau: yellow; Pacmanus: turquoise; Susu: blue and Woodlark: purple). The first four components shown here explain 31.2%, 25.4%, 18.1%, and 16.5% of the variance, respectively. The second principal component seems to be linked mildly with locality.

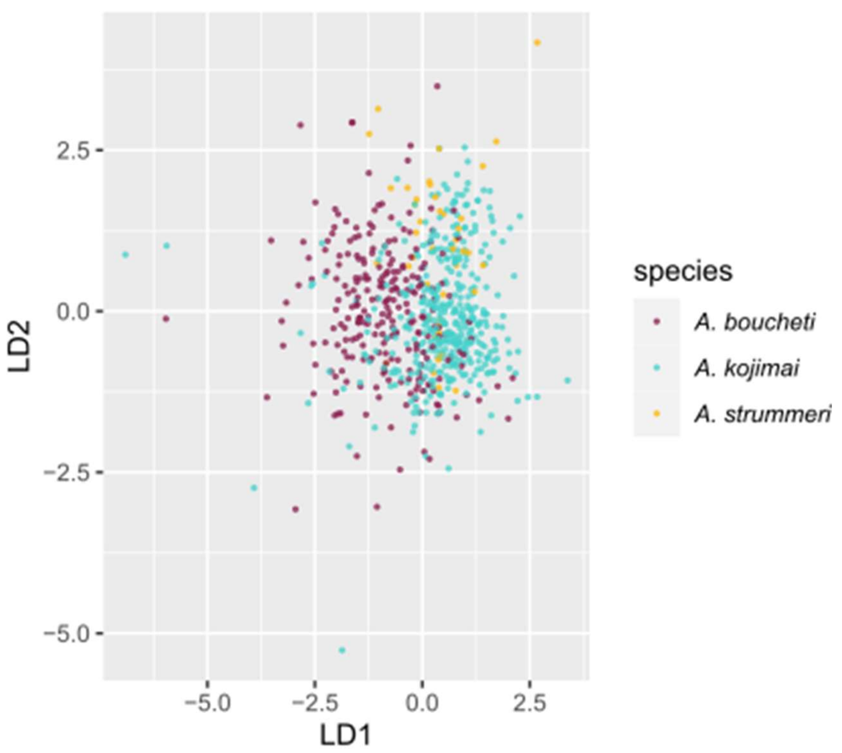

Figure S7. Linear discriminant analysis of species identity based on five transformed morphometric variables (see main text) for *Alviniconcha boucheti* (purple, *n*=247 ind.), *A. kojimai* (turquoise, *n*=409), and *A. strummeri* (orange, *n*=44). The combination of linear discriminants only allows a partial reclassification of species (75%).

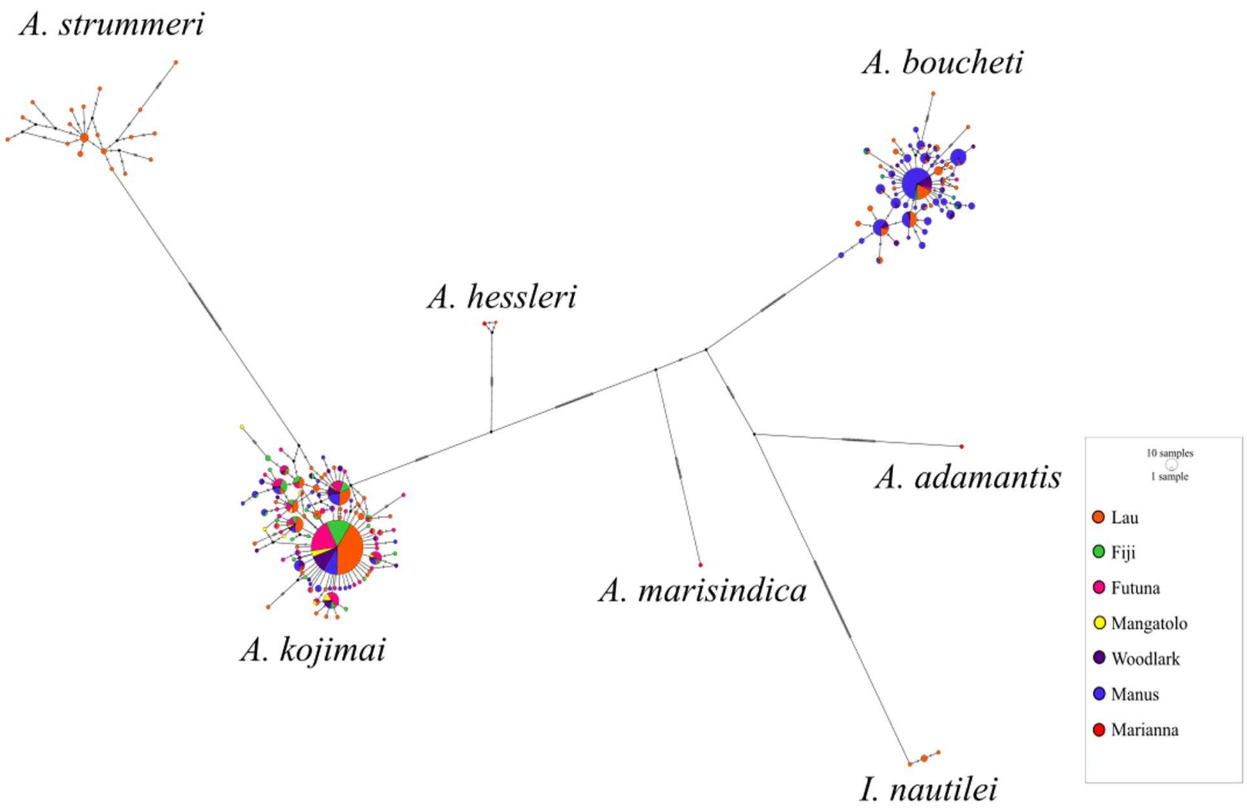

Figure S8. Haplotype network of 722 individuals of *Alviniconcha* spp for *Cox1* partial mitochondrial sequence rooted with *Ifremeria nautilei* (n= 4). Circles represent individual haplotypes, while circle size is proportional to haplotype frequency. The traits on the branches indicate the number of mutations between haplotypes. Orange: Lau Basin; Green: North-Fiji Basin, Pink: volcanic zone of Futuna, Yellow: Mangatolo site, Purple: Woodlark Basin, Blue: Manus Basin and Red: Marianna Trench.

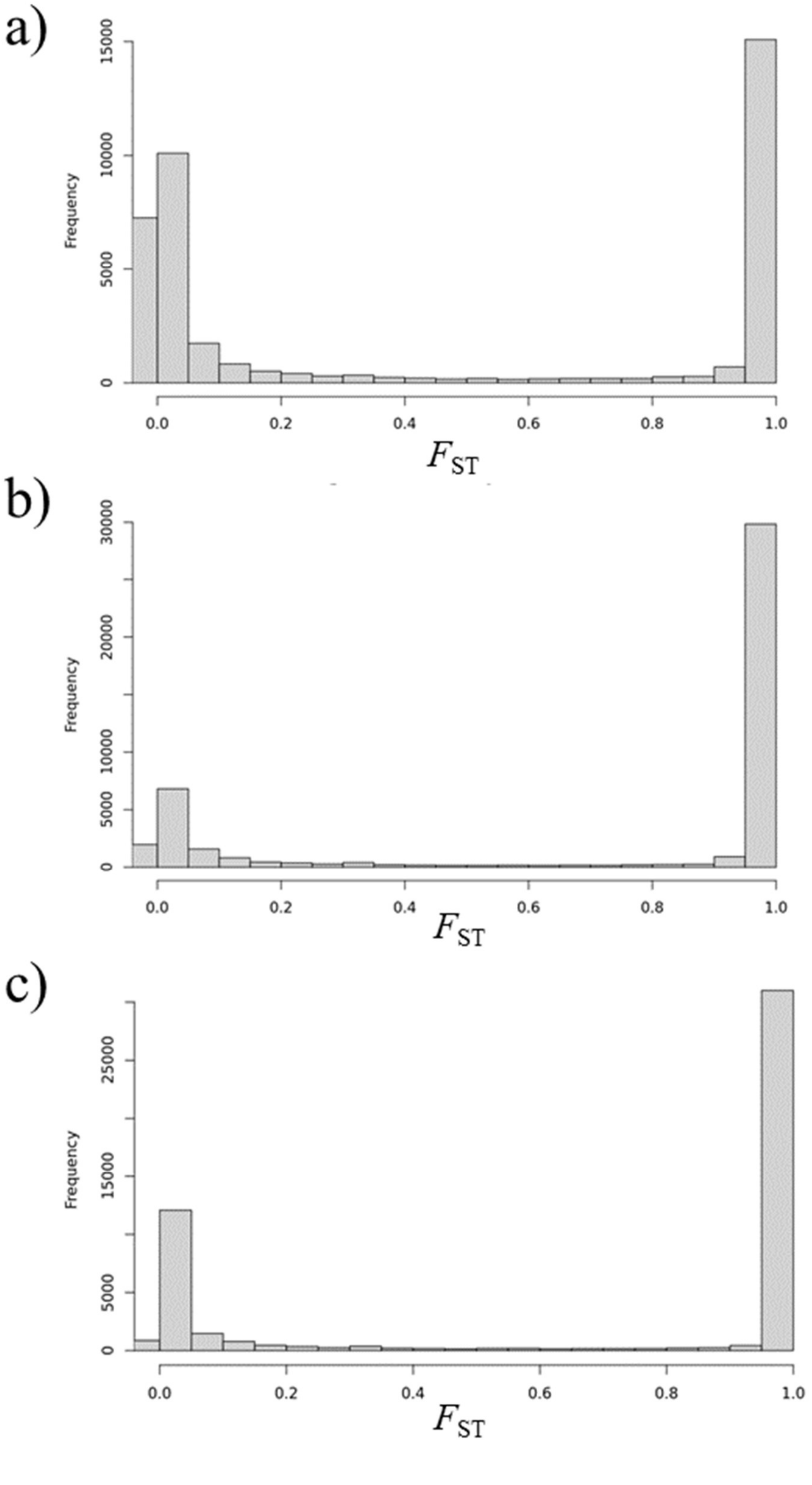

Figure S9. Distribution of F_{ST} values by species pair, a) *A. kojimai/A. strummeri* on 39 533 SNP, b) *A. boucheti/A. strummeri* on 45 514 SNP and c) *A. kojimai/A. boucheti* on 50 093 SNP.

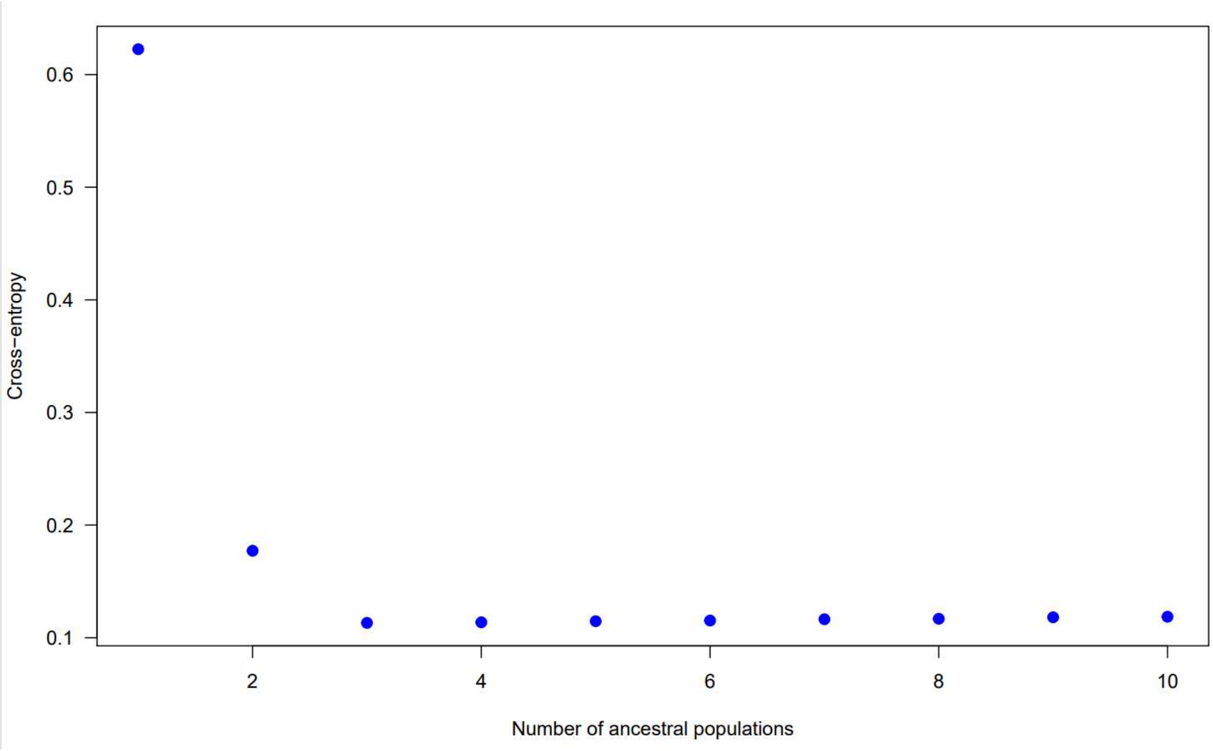

Figure S10. Values of the cross-entropy criterion as a function of the number of factors used in snmf runs.

Example of detailed parameter settings for *A. boucheti/A. kojimai* in DILS analysis:

infile: /beegfs/data/dils/8YVpfpJuxy/DILS_fichier.fasta

region: noncoding

nspecies: 2

nameA: boucheti

nameB: kojimai

nameOutgroup: NA

useSFS: 1

lightMode: FALSE

config_yaml: /beegfs/data/dils/8YVpfpJuxy/8YVpfpJuxy.yaml

timeStamp: 8YVpfpJuxy

population_growth: variable

modeBarrier: beta

max_N_tolerated: 0.2

Lmin: 144

nMin: 12

mu: 1e-08

rho_over_theta: 0.1

N_min: 100

N_max: 1000000

Tsplit_min: 100

Tsplit_max: 1000000

M_min: 0.4

M_max: 20
